# How to build a lichen: from metabolite release to symbiotic interplay

**DOI:** 10.1111/nph.18780

**Published:** 2023-03-04

**Authors:** Gregor Pichler, Lucia Muggia, Fabio Candotto Carniel, Martin Grube, Ilse Kranner

**Affiliations:** ^1^ Department of Botany University of Innsbruck Sternwartestraße 15 6020 Innsbruck Austria; ^2^ Department of Life Sciences University of Trieste Via L. Giorgieri 10 34127 Trieste Italy; ^3^ Institute of Biology University of Graz Holteigasse 6 8010 Graz Austria

**Keywords:** extracellular, fungus, metabolite, mycobiont, photobiot, polyol, signalling, symbiosis

## Abstract

Exposing their vegetative bodies to the light, lichens are outstanding amongst other fungal symbioses. Not requiring a pre‐established host, ‘lichenized fungi’ build an entirely new structure together with microbial photosynthetic partners that neither can form alone. The signals involved in the transition of a fungus and a compatible photosynthetic partner from a free‐living to a symbiotic state culminating in thallus formation, termed ‘lichenization’, and in the maintenance of the symbiosis, are poorly understood. Here, we synthesise the puzzle pieces of the scarce knowledge available into an updated concept of signalling involved in lichenization, comprising five main stages: (1) the ‘pre‐contact stage’, (2) the ‘contact stage’, (3) ‘envelopment’ of algal cells by the fungus, (4) their ‘incorporation’ into a pre‐thallus and (5) ‘differentiation’ into a complex thallus. Considering the involvement of extracellularly released metabolites in each phase, we propose that compounds such as fungal lectins and algal cyclic peptides elicit early contact between the symbionts‐to‐be, whereas phytohormone signalling, antioxidant protection and carbon exchange through sugars and sugar alcohols are of continued importance throughout all stages. In the fully formed lichen thallus, secondary lichen metabolites and mineral nutrition are suggested to stabilize the functionalities of the thallus, including the associated microbiota.

**Table:** 

	Contents	
	Summary	1362
I.	Introduction: from a ‘dual nature of lichens’ to a comprehensive one	1362
II.	Lichenization: transition from a free‐living to a symbiotic state	1365
III.	From arabitol to zeatin: a gradual shift in the contribution of metabolites from beginning to completion of lichenization?	1366
IV.	The thallus: a fine‐tuned symbiotic interplay supporting nutrition, tolerance of irradiation and desiccation, and reproduction	1372
V.	Outlook	1374
	Acknowledgements	1374
	References	1375

‘The whole is more than the sum of its parts’

Attributed to Aristotle

## I. Introduction: from a ‘dual nature of lichens’ to a comprehensive one

Mutualistic or self‐sustaining symbioses usually emerge in evolution through selection of novel pathways or phenotypes (Douglas, 2014). Indeed, the symbiotic stage often looks completely different from what the symbiotic partners produce in axenic solitude. This is particularly so with lichens, where the knitwork of fungal hyphae produces a unique miniature glasshouse to grow algae – a structure that surpasses most other vegetative fungal mycelia by the complexity of its organisation. Unsurprisingly, lichens were the first discovered case of symbioses, after they were considered as an own lower plant‐like group with unclear affiliation, although they were probably not the first to reach dry grounds in evolutionary history (Nelsen *et al*., 2020). Five‐hundred to 450 million years ago (Ma), the colonization of terrestrial habitats – when the descendants of streptophyte algae evolved into the first land plants – was a major transition on Earth (Becker, 2013), and the ability of plants to form symbiotic associations with beneficial, mycorrhizal fungi was one of the critical innovations required (Delaux *et al*., 2015). With the first undisputed appearance of lichens in the Lower Devonian, *c*. 415 Ma (Selosse & Le Tacon, 1998; Honegger *et al*., 2013), the lichen symbiosis could be viewed as a second, alternative way of conquering the land, when the Chlorophyta, together with the Ascomycota, evolved the first lichen thalli. Since then, lichens have massively diversified (Selosse & Le Tacon, 1998; Selosse *et al*., 2015), and their symbiotic lifestyle seems to be a requirement for sexual reproduction of the involved fungus, also implying that the signalling framework required to synthesize the symbiotic state is likewise conserved.

**Fig. 1:**
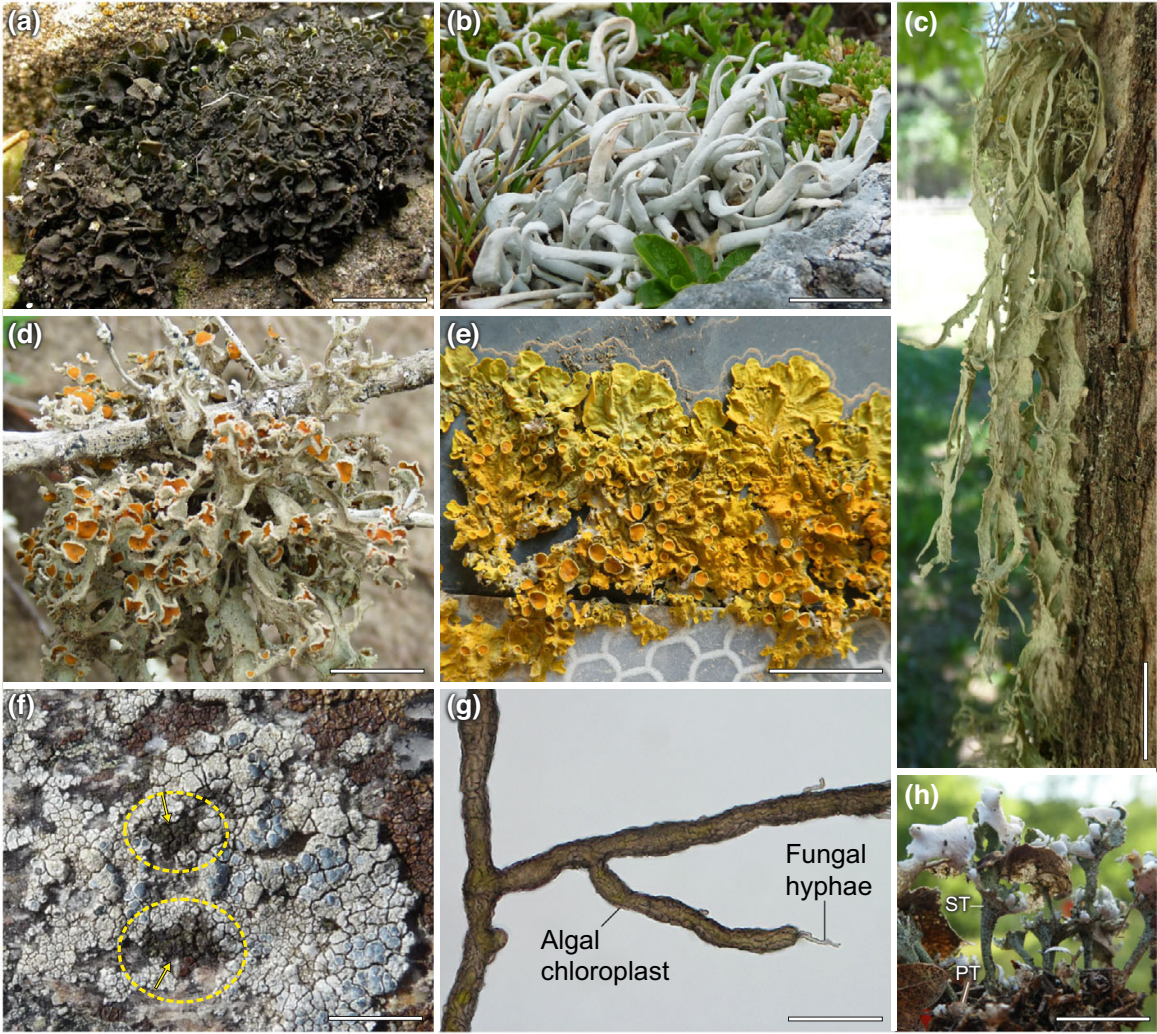
Lichen thallus growth forms. (a) *Collema crispum* (gelatinose, foliose/shrubby), (b) *Thamnolia vermicularis* (fruticose, with wormlike branches), (c) *Ramalina fraxinea* (fruticose/shrubby, hanging); (d) *Seirophora villosa* (fruticose/shrubby), (e) *Xanthoria parietina* (foliose); (f) *Lecanora rupicola* (crustose, epilithic) infected by the lichenicolous lichen *Rimularia insularis* (brown patches encircled by yellow dashed lines; yellow arrows), (g) *Cystocoleus ebeneus* (filamentous; arrows indicating the melanised fungus enwrapping *Trentepohlia* sp. filaments), (h) *Cladonia verticillata* (fruticose, arrows indicating the squamulose primary thallus (PT) and the erect podetia of the secondary thallus (ST)). Bars: (a, d, e, h) 1 cm; (b, c) 2 cm; (f) 10 cm; (g) 0.5 mm.

The ‘dual nature’ of lichens, first described by Schwendener, was initially interpreted as controlled parasitism, where an alga is ‘enslaved by a fungus and compelled into its service’ (Schwendener, 1869), and first attempts to culture them *in vitro* started soon after (Bornet, 1872). Nowadays, lichens are regarded as self‐sustaining ecosystems formed by the interaction of an exhabitant fungus, called ‘mycobiont’, forming a symbiosis with extracellular microbial photosynthetic partners, termed ‘photobiont’, and an indeterminate number of other microscopic organisms (Hawksworth & Grube, 2020). About 19 400 mycobiont species in 115 families have been identified, mostly Ascomycota, and a growing number of Basidiomycota species as their main fungal symbionts (Lücking *et al*., 2016). Considerably fewer species (*c*. 120) of unicellular green algae in the Chlorophyta are the known photobionts of an estimated 85% of lichens. Cyanobacteria are photobionts of another 10% of lichens. Around 3% are tripartite symbioses, in which both green algae (‘chlorobionts’) and cyanobacteria (‘cyanobionts’) co‐exist, whereby the cyanobacteria are usually compartmented in the so‐called ‘cephalodia’ within the thallus, and some lichen photobionts are yet to be described (Honegger, 2012). Furthermore, ‘lichen parasites’ or ‘lichenicolous fungi’ are known as specific fungal colonizers of lichen thalli, some of which – the ‘lichenicolous lichens’ – even form their own thallus structures (Lawrey & Diederich, 2003). Recent research illuminated the diversity of other micro‐organisms, that is the lichen microbiota (Grube & Berg, 2009; Muggia *et al*., 2016; Spribille *et al*., 2016; Spribille, 2018). Nevertheless, the mycobiont constitutes the main proportion of lichen biomass and, with very few exceptions, builds up the structures of the lichen thallus, shaping the various growth forms (Fig. 1) of lichens.

**Fig. 2:**
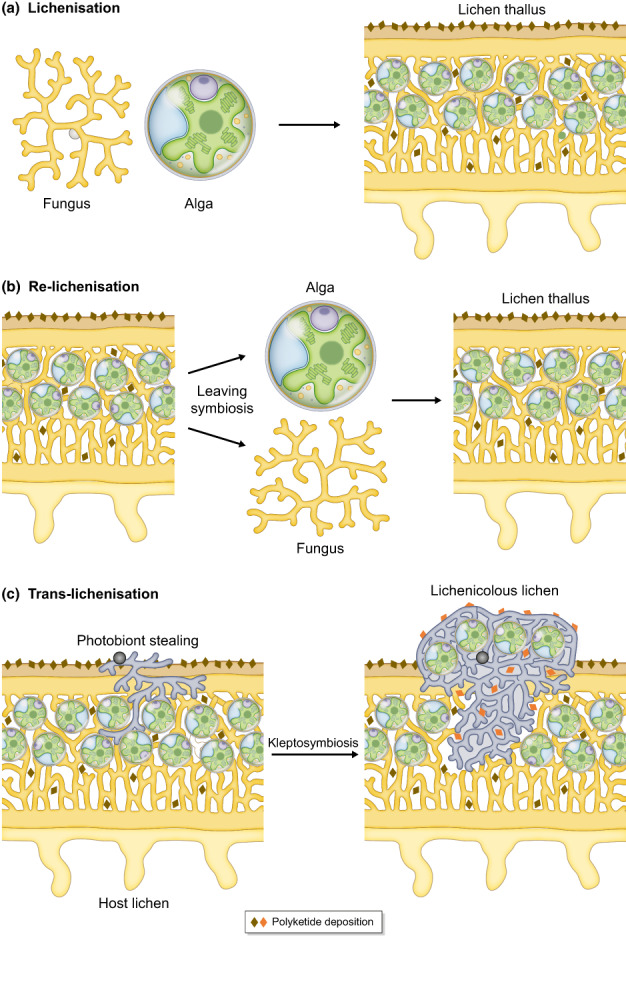
Proposed concept of lichenization, re‐lichenization and trans‐lichenization. (a) Lichenization – at an evolutionary time scale – defined as the transition of a free‐living fungus and its future microbial photobionts, green microalga and/or cyanobacteria, culminates in the formation of a lichen thallus, a self‐sustaining symbiosis that eventually comprises a plethora of other micro‐organisms in the lichen microbiota. (b) Re‐lichenization is referred to cases, in which the primary symbionts leave the symbiosis followed by building a new thallus. This occurs in nature, and can be achieved experimentally under laboratory conditions, from which most of our current knowledge on lichenization has been obtained. (c) Trans‐lichenization is shown for, although not restricted to, lichenicolous lichens, which capture photobionts from other lichens. This photobiont stealing by lichen‐forming fungi and establishment of yet another lichen thallus is proposed to be termed kleptosymbiosis.

The primary lichen myco‐ and photobionts can reproduce vegetatively by tiny structures containing the lichen‐forming fungi and their photobionts present in the parental thallus, but free‐living stages of the prospective symbionts are also found in nature (Tschermak‐Woess, 1978; Bubrick *et al*., 1984). Fungal dispersal units may be developed meiotically or mitotically. As no active structures for algal dispersal are known to occur in lichen thalli, photobionts are thought to be released from an established thallus, for example due to mechanical disruption, fungal decay or passage through feeding animals (Peksa *et al*., 2022). The synthesis of a lichen thallus from a free‐living state of compatible fungi and algae (cyanobacteria) has been referred to as ‘lichenization’ or ‘re‐lichenization’ (Sanders & Lücking, 2002), an event infrequently gained and also lost in the Ascomycota (Lutzoni *et al*., 2001). Alternatively, and instead of synonymous use, the term lichenization could be reserved for the very first transition of a naïve free‐living fungus and its future microbial photobionts, through evolution, into a self‐sustaining thallus, and re‐lichenization to cases in which mycobionts and photobionts were released or separated from existing lichen thalli, either in nature or in the laboratory, and then re‐synthesised into a thallus. Furthermore, perhaps more frequently than expected, free‐living lichen‐forming fungi may capture compatible green algae from various stages of already existing lichen thalli, and lichenicolous lichens appear to specialize on such photobiont stealing. We suggest the term ‘trans‐lichenization’ to describe the capture of already lichenized algae by another mycobiont, and the newly formed thallus as ‘kleptosymbiosis’ (Fig. 2). These cases merit differentiating, as previously lichenized algae already experienced transcriptomic (re)‐programming, as deduced from the changing ultrastructural details (De Los *et al*., 2002).

The specific processes involved in re‐programming through trans‐lichenization may explain why lichenicolous lichens are generally very selective for their hosts. In addition, there are also some examples of ‘lichen evolution in progress’. For example, *Schizoxylon* sp. can either live alone, saprophytically, or form a loose symbiosis with poorly distinguishable thalli together with *Coccomyxa* sp. photobionts (Muggia *et al*., 2011), and some of the filamentous *Trentepohlia* species can occur in free‐living colonies, be lichen photobionts (Fig. 1g) or parasites (Hametner *et al*., 2014). Most knowledge gained about lichenization is derived from laboratory studies, that is re‐lichenization *sensu stricto*, but below we continue to use the terms lichenization and re‐lichenization synonymously, in line with the literature available.

## II. Lichenization: transition from a free‐living to a symbiotic state

### 1. The stages of lichenization

**Fig. 3:**
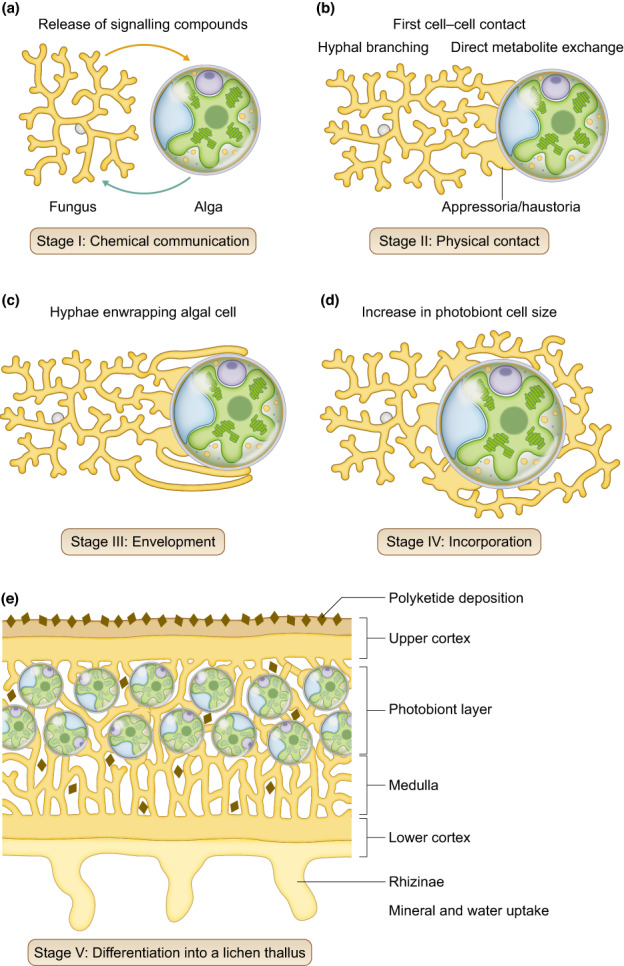
The stages of lichenization. (a) The ‘pre‐contact stage’, before fungus and alga are in physical contact, is characterized by increased hyphal branching and mucilage production by the fungus, and release of molecules by the fungus and the alga into the environment, supporting mutual recognition. (b) In the ‘contact stage’, appressoria and haustoria are formed by the fungus, physically connecting mycobiont hyphae and photobiont cells, enabling direct exchange of metabolites. (c) In the ‘envelopment stage’, fungal biomass increases, more appressoria and haustoria are formed, and hyphae engulf the photobiont cells. (d) In the ‘incorporation’ stage, fungal hyphae and photobiont cells form undifferentiated aggregations and algal cell size increases. (e) The final ‘differentiation stage’ comprises the differentiation into a lichen thallus. See section ‘II. Lichenization: transition from a free‐living to a symbiotic state’ for details and citations.

A synthesis of topical papers (Trembley *et al*., 2002; Joneson & Lutzoni, 2009; Joneson *et al*., 2011; Meeßen *et al*., 2013; Meeßen & Ott, 2013; Athukorala *et al*., 2014; Insarova & Blagoveshchenskaya, 2016; Kono *et al*., 2020) on lichenization of fruticose or squamulose lichens, mostly in the orders Lecanorales or Teloschistales, reveals that lichenization is currently thought to comprise several steps. These steps are summarized in Fig. 3 and envisaged to blend smoothly from one into the other, that is without abrupt shifts. In the ‘pre‐contact stage’, the fungus and alga are in close proximity, but without physical contact. It is assumed that chemical signalling supports mutual recognition in this phase, followed by increased mucilage production and hyphal branching of the fungus and growth towards the algae. This first, visually detectable indication of a mycobiont recognizing a potential photobiont is followed by the ‘contact stage’, during which mycobiont hyphae establish physical contact with photobiont cells, typically through appressoria or different types of haustoria (Honegger, 1986, 2012). In the ‘envelopment stage’, the fungal hyphae enwrap the photobiont cells almost completely. In the ‘incorporation stage’, hyphae and photobiont cells form an undifferentiated aggregation of biomass, and the ‘differentiation stage’ entails organization into a (commonly stratified) thallus, which can only be achieved by compatible myco‐ and photobionts (Meeßen & Ott, 2013). In the fully formed thallus, the mycobiont controls the development of photobiont cell division and growth, as well as regulating photobiont cell size and ultrastructure (Hill, 1989, 1993). It is likely that this control by the fungus commences early during lichenization.

It is not precisely known how long it takes to complete lichenization after the first sensing among the partners in nature, but on exposed plastic leaves in a rain‐forest lichen symbioses established within relatively short periods of time, in the range of months (Sanders, 2002). In the laboratory, at least a few months up to several years of co‐culture of previously isolated primary symbionts are required to achieve re‐lichenization into fully formed lichen thalli (e.g. Stocker‐Wörgötter & Türk, 1991; Yoshimura *et al*., 1993; Stocker‐Wörgötter, 2001a,b; Kono *et al*., 2020). Specific abiotic factors such as cycles of desiccation and rehydration promote *in vitro* lichenization (Stocker‐Wörgötter, 2001b). A need for desiccation also agrees with the suggestion that the ability to tolerate desiccation and extensive irradiation is a prerequisite for, and driving, lichen evolution (Kranner & Lutzoni, 1999).

### 2. Molecular crosstalk accompanies mutual recognition of the prospective symbionts

**Fig. 4:**
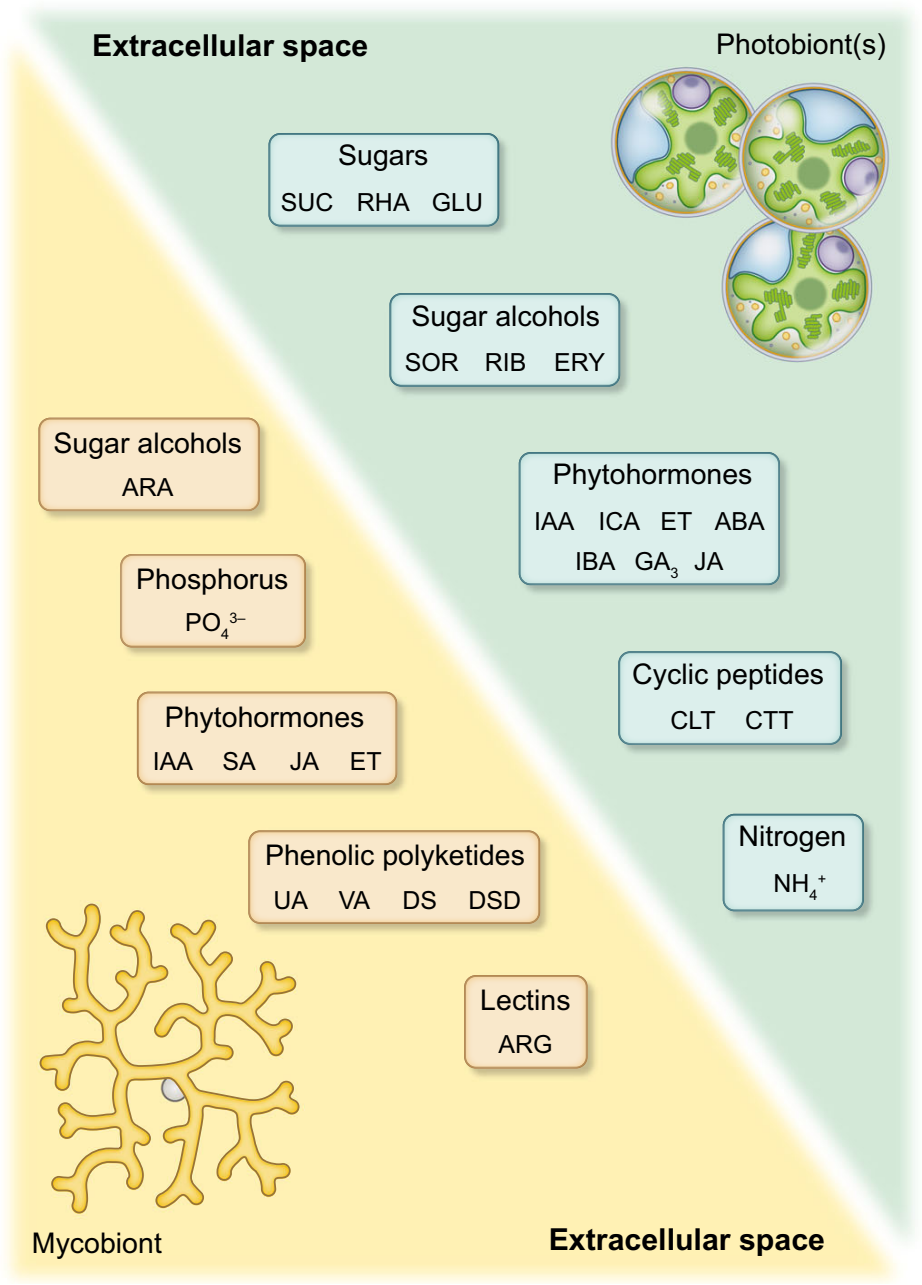
Chemical compounds released by isolated lichen myco‐ and photobionts into their environment. Photobionts have been shown to release the sugars glucose (GLU), rhamnose (RHA) and sucrose (SUC), the sugar alcohols sorbitol (SOR), ribitol (RIB), and erythritol (ERY), the phytohormones indole‐3‐acetic acid (IAA) and its precursor indole‐carbaldehyde (ICA), indole‐3‐butyric acid (IBA), abscisic acid (ABA), gibberellin A3 (GA_3_), jasmonic acid (JA), and ethylene (ET), and the cyclic peptides cyclo‐(l‐leucyl‐l‐tyrosyl) (CLT), and cyclo‐(l‐tryptophyl‐l‐tryptophyl) (CTT). Mycobionts release the sugar alcohol arabitol (ARA), phosphate (PO_4_^3−^), the phytohormones IAA, salicylic acid (SA), JA, and ET, lectins with arginase (ARG) activity, and the phenolic polyketides usnic acid (UA), vulpinic acid (VA), and various depsides (DS), and depsidones (DSD). See text and Tables 1 and 2 for details and references.

Ahmadjian & Jacobs (1981) put forward that a fungus recognizes a prospective photobiont by the shape of the algal cells, referred to as ‘thigmotropism hypothesis’. Joneson & Lutzoni (2009) experimentally achieved mycobiont differentiation only with compatible lichen‐forming algae rather than inanimate objects such as glass beads. These results, together with the finding that between 11 and 28% of mycobiont and photobiont genes are already upregulated during the ‘pre‐contact’ and the ‘contact stage’, supported the ‘signalling hypothesis’ based on chemical signalling between symbionts before physical contact (Joneson *et al*., 2011). We now know that lichenization is accompanied by regulation of gene expression in each partner involved, and this will induce downstream proteomic and metabolomic re‐arrangements (Trembley *et al*., 2002; Joneson *et al*., 2011; Armaleo *et al*., 2019; Kono *et al*., 2020; Sveshnikova & Piercey‐Normore, 2021). For example, genes encoding signal transduction components are already upregulated in the contact stage in both, mycobiont and photobiont, as elegantly demonstrated for the model lichen, *Cladonia grayi*, and its *Asterochloris glomerata* photobiont: specifically, genes encoding extracellular hydrolases and membrane transport proteins are upregulated in both prospective symbionts, and ammonium and ribitol transporters in the fungus only (Armaleo *et al*., 2019). Furthermore, the microbiota may influence lichen metabolism, for example phytohormone, vitamin, carbohydrate, lipid and nitrogen metabolism, thereby contributing to the fine‐tuning of the symbiotic relationship of lichens, especially when challenged by biotic and abiotic stress factors (Erlacher *et al*., 2015; Cernava *et al*., 2017). Considering these transcriptional responses, symbiotic interaction must be accompanied by molecular crosstalk, that is the intricate process by which signal transduction pathways are affected by components of others. Importantly, crosstalk requires chemical compounds to be released into the extracellular matrix and perceived by the symbiotic partner. Signals can then be processed within the cell to induce downstream effects on the transcriptome, proteome and metabolome, and thus physiology and morphology of the symbiotic partners. However, our knowledge of compounds that affect signal transduction pathways during lichenization is still in its infancy. Next, we review reports on compounds released by lichen mycobionts and photobionts into the extracellular space (see Fig. 4 for overview), addressing potential roles of primary and secondary metabolites from the early stages of lichenization up to the fully formed thallus, appreciating that all are important for the development of a functional thallus.

## III. From arabitol to zeatin: a gradual shift in the contribution of metabolites from beginning to completion of lichenization?

### 1. Elicitors in the pre‐contact and contact stage

Making contact depends on prior recognition between the symbionts, and evidence is emerging that elicitors interacting with the cell wall are involved (Wang *et al*., 2014; Díaz *et al*., 2016). Lectins, although they are not receptors *per se*, are proteins that bind to specific carbohydrate residues and mediate attachment of micro‐organisms such as fungi, bacteria, microalgae or viruses to target cells (Singh & Walia, 2014; Romero *et al*., 2021). When lectins recognize their target carbohydrates embedded in the outer surface of cell walls, cells are bound and lectins can activate enzyme cascades involved in signalling processes, inducing metabolic and physiological rearrangements (Molina & Vicente, 1995; Alen'kina *et al*., 2014). Using the model lichen, *Xanthoria parietina*, Bubrick & Galun (1980) showed that mycobiont proteins can bind to acidic polysaccharide residues on the outer cell wall of five photobiont species isolated from Teloschistaceae lichens, but not to photobionts from other lichen families, suggesting that only compatible photobionts are targeted. A fungal lectin with arginase activity binding to polygalactosylated urease was found in the photobiont cell wall, and glycosylated urease production was limited to periods of algal cell division (Sacristán *et al*., 2006). Arginase secretion emerges as a key factor in the recognition of compatible algae, and fungal lectins appear to be involved in the differentiated recognition of green algae and cyanobacteria, leading to the formation of chlorolichens and cyanolichens, respectively (Legaz *et al*., 2004; Vivas *et al*., 2010), even though the precise recognition mechanisms remain to be elucidated. Recruitment of *Nostoc* sp. cells by *Peltigera canina* seems to be induced by glycosylated arginase, the adhesion of which to the hyphal surface lectins is achieved by contraction‐relaxation episodes of the cytoskeleton (Díaz *et al*., 2011).

Moreover, a cyclic peptide, cyclo‐(l‐tryptophyl‐l‐tryptophyl) was found in the exudates of four different *Trebouxia* sp. photobionts isolated from the lichens *Gyalolechia bracteata* (syn. *Fulgensia bracteata*), *Gyalolechia fulgens*, *Toninia sedifolia* and *Rusavskia elegans* (syn. *Xanthoria elegans*). *Asterochloris* sp. isolated from *Romjularia lurida* (syn. *Lecidea lurida*), released cyclo‐(l‐leucyl‐l‐tyrosyl) in addition to cyclo‐(l‐tryptophyl‐l‐tryptophyl). When applied exogenously to the *Gyalolechia bracteata* mycobiont, cyclo‐(l‐tryptophyl‐l‐tryptophyl) triggered ascospore germination (Meeßen *et al*., 2013). Therefore, the sparse evidence suggests that in the precontact and the contact stage, fungal lectins – as readers of the sugar code – elicit contact and are involved in recognizing compatible photobionts, and algal metabolites such as cyclic peptides released into the extracellular environment attract potential mycobionts, and affect fungal sexual reproduction.

### 2. Sugars and sugar alcohols from the precontact stage through to the fully established lichen thallus

**Fig. 5:**
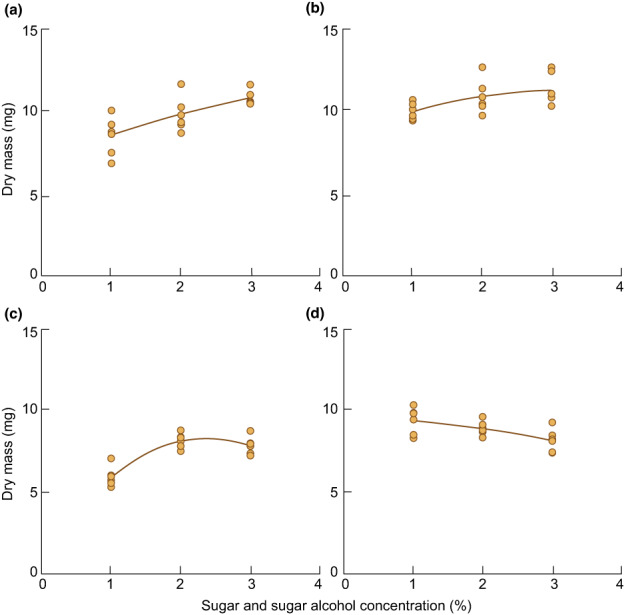
Sugar and sugar alcohol‐dependent cumulative growth of the mycobiont isolated from *Xanthoria parietina*. The response of fungal growth on solid Lilly‐Barnett Medium (LBM) supplemented with either 1, 2 or 3% of (a) d‐glucose (*R* ^2^ = 0.55), (b) d‐mannitol (*R* ^2^ = 0.31), (c) d‐arabitol (*R* ^2^ = 0.80) or (d) ribitol (*R* ^2^ = 0.41), as carbon source was assessed after 8 wk (*n* = 6 biological replicates); each dot represents a single value, dotted lines show trend curves. Data were re‐analysed from fig. 5 in Pichler *et al*. (2021).

Within the intact thallus, and also when isolated and grown axenically, myco‐ and photobionts release sugars and sugar alcohols into the extracellular space (Table 1). Sugar alcohols containing multiple hydroxyl groups are often referred to as ‘polyols’, but this term is somewhat ambiguous as it is also used in polymer chemistry, so that we prefer the term sugar alcohols. These molecules appear to be early attractants for the carbohydrate‐loving mycobionts *in spe*. Release of sugar alcohols was reported mainly for photobionts, disregarding release by the mycobiont, with one exception (Wang *et al*., 2014), perhaps reflecting an anticipated role of the photobiont as the main nurturer. When isolated from the thallus and then co‐cultivated with its mycobiont, the photobiont *Diplosphaera chodatii* released sorbitol, glucose and sucrose, and the mycobiont incorporated these compounds using specific transporters. Sorbitol and sucrose can be then converted into fructose and glucose, and enter fungal glycolysis. However, in the absence of the mycobiont, sorbitol, glucose and sucrose were not found in exudates of axenically cultured *Diplosphaera chodatii* photobionts, so it appears that a stimulus from the mycobiont is required to trigger the release of these compounds (Wang *et al*., 2014). If this assumption is correct, it follows that chemical communication between the myco‐ and photobionts through sugars and sugar alcohols contains push‐pull elements, that is transport from sink to source. Fungal stimuli could involve the actions of yet to be determined members of fungal carbohydrate‐active enzymes (CAZymes), which may enhance the permeability of algal cell walls. Furthermore, the isolated photobionts of *Gyalolechia bracteata*, but not free‐living green algae, also release rhamnose, which may be specific for lichen‐forming algae (Meeßen *et al*., 2013). Interestingly, unlike cyclo‐(l‐tryptophyl‐l‐tryptophyl), rhamnose suppresses ascospore germination (Meeßen *et al*., 2013). Moreover, exogenous treatment with sugars and sugar alcohols affects the growth of cultured lichen mycobionts. For example, ribitol stimulated growth of the *Ramalina farinacea* and *Ramalina fastigiata* mycobionts (Wang *et al*., 2009), and mannitol and glucose that of the *Xanthoria parietina* mycobiont, although in the latter growth rates decreased with increasing ribitol concentrations (Pichler *et al*., 2021). Thus, the preference for specific sugars and sugar alcohols as carbon sources may depend on mycobiont species, and sugars and sugar alcohols can affect mycobiont growth in a concentration‐dependent manner (Fig. 5). Furthermore, ribitol also suppressed and mannitol enhanced mycobiont secondary metabolite production in axenic mycobiont cultures of *Rusavskia elegans* (Brunauer *et al*., 2007). Radioactively labelled [^3^H] mannitol can be incorporated into the cell walls of isolated mycobionts, but not into the cell walls of other, free‐living fungi (Galun *et al*., 1976). This points to the existence of specific mannitol and other sugar alcohol transporters in lichen mycobionts (Yoshino *et al*., 2019), but it remains to be tested whether mannitol incorporation into cell walls is a key feature required for lichenization. Based on the finding that many genes encoding CAZymes were found in 46 representative mycobiont genomes, it was suggested, albeit not experimentally proven, that mycobionts could metabolize algal carbohydrates and cell wall components, or carbon from other sources (Resl *et al*., 2022).

**Table 1:** Sugars and sugar alcohols released by lichen myco‐ and photobionts.

***Mycobiont***	Sugars	Sugar alcohols	References
*Photobiont*			
***Coccocarpia* sp.**	nd	nd	Richardson *et al*. (1968)
*Scytonema *sp.	Glucose*	nd	Hill & Smith (1972)
***Collema auriculatum***	nd	nd	
*Nostoc* sp.	Glucose*	nd	Richardson *et al*. (1968)
***Dermatocarpon hepaticum***	nd	nd	Richardson *et al*. (1968)
*Myrmecia* sp.	nd	Ribitol*	Hill & Smith (1972)
***Dermatocarpon miniatum*, *D. fluviatile***	nd	nd	Hill & Smith (1972)
*Hyalococcus* sp.	nd	Sorbitol*	Richardson *et al*. (1968)
***Endocarpon pusillum***	nd	nd	
*Diplosphaera chodatii*	Glucose, Sucrose	Sorbitol	Wang *et al*. (2014)
***Fulgensia bracteata***	nd	nd	
*Trebouxia* sp.	Rhamnose	nd	Meeßen *et al*. (2013)
***Gyalecta cupularis***	nd	nd	
*Trentepohlia* sp.	nd	Erythritol*	Richardson *et al*. (1968)
***Lecanactis stenhammarii***	nd	nd	
*Trentepohlia* sp.	nd	Erythritol*	Richardson *et al*. (1968)
***Lecanora conizaeoides***	nd	nd	
*Trebouxia* sp.	nd	Ribitol*	Richardson *et al*. (1968)
***Lepraria incana*, *L. chlorine***	nd	nd	
*Trebouxia* sp.	nd	Ribitol*	Hill & Smith (1972)
***Lichina pygmaea***	nd	nd	
*Calothrix* sp.	Glucose*	nd	Richardson *et al*. (1968)
***Lobaria amplissima***	nd	nd	
*Coccomyxa* sp., (*Nostoc* sp.)	(Glucose)*	Ribitol*	Richardson *et al*. (1968)
***Lobaria laetevirens***	nd	nd	
*Myrmecia* sp.	nd	Ribitol*	Richardson *et al*. (1968)
***Lobaria scrobiculata***	nd	nd	
*Nostoc* sp.	Glucose*	nd	Richardson *et al*. (1968)
***Peltigera polydactyla*, *P. canina***	nd	nd	Drew & Smith (1967)
***P. horizontalis*, *P. polydactyla***	nd	nd	Richardson *et al*. (1968)
*Nostoc* sp.	Glucose*	nd	Hill & Smith (1972)
***Peltigera aphthosa***	nd	nd	Hill & Smith (1972)
*Coccomyxa* sp., (*Nostoc* sp.)	(Glucose)*	Ribitol*	Richardson *et al*. (1968)
***Pseudevernia furfuracea*, *P. saxatilis***	nd	nd	
*Trebouxia* sp.	nd	Ribitol*	Richardson *et al*. (1968)
***Porina lectissima***	nd	nd	
*Trentepohlia* sp.	nd	Erythritol*	Hill & Smith (1972)
***Ramalina crassa*, *R. subbreviuscula***	nd	nd	
*Trebouxia* sp.	nd	Ribitol*	Komiya & Shibata (1971)
***Ramalina yasudae***	nd	Arabitol	Kosugi *et al*. (2013)
*Trebouxia* sp.	nd	Ribitol	
***Roccella montagnei*, *R. fuciformis***, ***R. phycopsis***	nd	nd	Hill & Smith (1972)
*Trentepohlia* sp.	nd	Erythritol*	Richardson *et al*. (1968)
***Solorina saccata***	nd	nd	
*Coccomyca* sp., (*Nostoc* sp.)	(Glucose)*	Ribitol*	Richardson *et al*. (1968)
***Sphaerophorus globosus***	nd	nd	
*Trebouxia* sp.	nd	Ribitol*	Hill & Smith (1972)
***Sticta fuliginosa***	nd	nd	
*Nostoc* sp.	Glucose*	nd	Richardson *et al*. (1968)
***Umbilicaria pustulata***	nd	nd	
*Trebouxia* sp.	nd	Ribitol*	Richardson *et al*. (1968)
***Xanthoria aureola*, *X. calcicola***	nd	nd	Richardson *et al*. (1968)
*Trebouxia* sp.	nd	Ribitol*	Hill & Smith (1972)
			Lines *et al*. (1989)

Sugars and sugar alcohols exchanged between lichen symbionts within the thallus assessed using radioactive labelled CO_2_ are marked with an asterisk, the rest were detected in exudates of isolated lichen myco‐ and photobionts. The third photosynthetic partners of tripartite lichens are listed in brackets; nd, not determined. Bold and regular text in the left column indicates mycobiont and photobiont species, respectively.

It follows that sugars and sugar alcohols released extracellularly by algae could be incorporated by fungi in the precontact stage, serving as attractants for future mycobionts. So far, no reports are available on the release, uptake or the effects of sugars and sugar alcohols on the fungal and algal transcriptomes during algal envelopment and incorporation by the fungus (stages III and IV in Fig. 3). Nevertheless, sugars and sugar alcohols are key carbon sources exchanged between the symbionts in intact lichen thalli, and therefore are likely pivotal for the symbiotic interplay between lichen photo‐ and mycobionts throughout lichenization.

In fully formed lichen thalli, sugars and sugar alcohols produced by photobionts are the main carbon sources for their mycobionts, as demonstrated by pioneering studies from the 1960s and 1970s. For example, ribitol, erythritol, sorbitol and glucose released by lichen photobionts are taken up by their corresponding mycobionts in many lichens (Richardson *et al*., 1968; Lines *et al*., 1989). The type of sugars and sugar alcohols provided by lichen photobionts to their mycobionts seems to reflect the phylogenetic status of the photobiont species. Early key studies revealed that glucose is the main photosynthate provided by *Nostoc* sp., ribitol by *Trebouxia*, and erythritol by *Trentepohlia* (Richardson *et al*., 1968; Hill & Smith, 1972). The current view is that glucose is the main photosynthate transported to fungal mycobionts by cyanobacterial photobionts, whereas the green lineage uses sugar alcohols. Specifically, tetritols, such as erythritol, appear to be the main sugar alcohols shunted to the fungal mycobionts by green microalgae in the Chlorophyceae and Trentepohliales, whereas other clades, including Prasiolales and Trebouxiales, use pentitols, such as ribitol (see Spribille *et al*., 2022; for summary).

Intriguingly, mycobionts can convert sugars and sugar alcohols produced by photobionts into other sugar alcohols (Eisenreich *et al*., 2011) and can even return them to their photobionts. For example, the *Xanthoria calcicola* mycobiont converts ribitol into mannitol, elegantly shown via ^13^C NMR analysis (Lines *et al*., 1989), and ribitol produced by the photobiont can be converted into arabitol and mannitol by *Ramalina* spp. mycobionts (Komiya & Shibata, 1971). Later, ribitol transport from the photobiont to the mycobiont, which can convert it into arabitol and return it back to the photobiont, was confirmed for *Ramalina yasudae* (Kosugi *et al*., 2013). Mannitol and arabitol are present in mycobiont hyphae of many species, but glycerol, volemitol and erythritol only in a few (Honegger *et al*., 1993). Generally, mycobionts apparently convert glucose into mannitol, and ribitol into arabitol and mannitol after import by the fungus, whereas erythritol may remain unchanged and moves only slowly between the symbionts (Richardson *et al*., 1968; Komiya & Shibata, 1971; Spribille *et al*., 2022).

Evidence suggests that d‐arabitol improves the photobiont's ability to dissipate excess light energy during desiccation (Kosugi *et al*., 2013), ribitol increase *F*_v_/*F*_m_, that is the photobiont's maximum quantum yield of photosystem II (Hájek *et al*., 2009a), and both, mannitol and ribitol, enhance the solubilisation of fungal enzymes during freezing events, providing cryoprotection (Fontaniella *et al*., 2000; Hájek *et al*., 2009b). Therefore, the complex symbiotic interplay within a lichen thallus involves provision of carbon from the photobiont to the mycobiont, but also mutual stimulation of fitness parameters such as photosynthetic performance of the photobiont and enhanced freezing tolerance of the mycobiont.

Moreover, the release of sugars and sugar alcohols also benefits the lichen microbiota. Glucose, ribitol and mannitol can be taken up by Alphaproteobacteria associated with lichen photobionts (Kono *et al*., 2017). Bacteria associated with the lichen *Leptogium puberulum* can catabolize degradation products of the lichen thallus, such as d‐cellobiose and N‐acetyl‐d‐glucosamine, mycobiont‐specific d‐mannitol and glucose‐containing carbohydrates of the photobiont (Grzesiak *et al*., 2021). Furthermore, lichenicolous fungi can metabolise mannitol and ribitol (Yoshino *et al*., 2020). In return, components of the microbiota, for example basidiomycete yeasts, may contribute polysaccharides to the lichen matrix (Tagirdzhanova *et al*., 2021). Therefore, sugars and sugar alcohols released by the primary lichen symbionts are likely attractants for other micro‐organisms to join the symbiosis. Lichen sugars and sugar alcohols may also influence the composition of lichen‐inhabiting bacteria, although chemical communication between the primary myco‐ and photobionts and lichen‐inhabiting micro‐organisms is not well‐understood (Grimm *et al*., 2021). In summary, sugars and sugar alcohols appear to be key molecules involved in the chemical crosstalk between lichen myco‐ and photobionts, from the precontact stage through to the fully established lichen thallus, with important roles for development, growth and fitness.

### 3. Phytohormones from the precontact stage through to the intact thallus

Phytohormones such as abscisic acid (ABA), auxins, brassinosteroids, cytokinins, ethylene, gibberellins, jasmonates, salicylic acid and strigolactones are pivotal signalling compounds in vascular plants, implicated in the regulation of plant growth, development and stress response (Santner *et al*., 2009). We use the widely accepted term ‘phytohormone’, but acknowledge that many phytohormones are derivates of simple metabolic compounds and are not exclusive to vascular plants, but are also produced by bacteria, algae, fungi and bryophytes (Costacurta & Vanderleyden, 1995; Decker *et al*., 2006; Tarakhovskaya *et al*., 2007; Chanclud & Morel, 2016). Therefore, phytohormones must have evolved before the vascular plants. Unsurprisingly, they also exist in lichens. Phytohormones are involved in the molecular crosstalk between organisms across plants, fungi, micro‐organisms and others, and hence, are inter‐ and multikingdom signalling compounds (Foo *et al*., 2019), whose roles are much better understood in mycorrhiza – the symbiotic associations between plants and beneficial, mycorrhizal fungi (Zygomycota, Glomeromycota, Ascomycota, Basidiomycota) – than in lichens. In arbuscular mycorrhiza, involving penetration of the host's cell walls by the fungus, strigolactones exuded by plants roots attract fungi and influence fungal morphology and metabolism (Parniske, 2008). Arbuscular mycorrhiza can be promoted by ABA, auxins and strigolactones, supressed by brassinosteroids, cytokinins and gibberellins (Pozo *et al*., 2015; Liao *et al*., 2018), and jasmonic acid and salicylic acid signalling can influence the expression of plant defence genes for plant pathogen resistance (Pozo & Azcón‐Aguilar, 2007). Ectomycorrhiza between mycorrhizal fungi and perennial, usually woody plants, is also regulated by phytohormones (Basso *et al*., 2020; Abdulsalam *et al*., 2021). For example, the formation of the Hartig net between root cortex and rhizodermis cells can be stimulated by a key auxin, indole‐3‐acetic acid (IAA), which apparently represents one of the most abundant phytohormones involved in ectomycorrhizae (Ek *et al*., 1983; Gay *et al*., 1994). However, many questions remain open about the role of phytohormones in lichens.

As for other compounds involved in lichenization, a prerequisite for phytohormones to affect lichenization is that they are released into the extracellular space of the prospective symbionts. The sparse evidence available suggests that this is the case (see Table 2a for summary). For example, isolated myco‐ and photobionts of the lichens *Cetraria islandica* and *Cladonia rangiferina* can release ethylene and its precursor 1‐aminocyclopropane‐1‐carboxylic acid (Schieleit & Ott, 1996). Indole‐3‐carbaldehyde, a precursor and degradation product of IAA (Bandurski *et al*., 1995; Gazarian *et al*., 1998), was present in the growth media of lichen‐forming *Trebouxia* spp. and suppressed fungal spore germination and hyphal growth of the *Gyalolechia bracteata* mycobiont (Meeßen *et al*., 2013). Indole‐3‐acetic acid was produced and extracellularly released by mycobionts and photobionts isolated from *Cladonia grayi*, *Tephromela atra* and *Xanthoria parietina* in a species‐ and (for photobionts) light‐dependent manner (Table 2b), while production and release of ABA, gibberellin A3, indole‐3‐butyric acid and jasmonic acid depended on photobiont species, and of salicylic acid and jasmonic acid on mycobiont species (Pichler *et al*., 2020a,b). Exogenously supplied IAA and indole‐3‐butyric acid stimulated growth of isolated mycobionts (Wang *et al*., 2009, 2010) and exogenous IAA treatment increased the water contents of isolated lichen photobiont cultures, although more research is needed to evaluate whether the vacuole, the cytoplasm, the cell wall or the intercellular water between the photobiont cells is responsible for the increased water contents (Pichler *et al*., 2020b). Therefore, these few reports provide evidence that phytohormones released by prospective symbionts are present in the extracellular space, where they could be mutually perceived, supporting lichenization from the precontact stage onwards.

**Table 2:** Evidence for extracellular phytohormone and cyclic peptide release by isolated lichen photo‐and mycobionts.

***Mycobiont***	Phytohormones	Cyclic peptides	References
*Photobiont*			
(a)			
***Cetraria islandica***	ET	nd	Schieleit & Ott (1996)
*Coccomyxa* sp.	ET	nd	
***Cladonia grayi***	IAA, JA	nd	Pichler *et al*. (2020b)
*Asterochloris glomerata*	IAA, ABA, JA	nd	Pichler *et al*. (2020a)
***Cladonia rangiferina***	ET	nd	Schieleit & Ott (1996)
*Trebouxia irregularis*	ET	nd	
***Fulgensia bracteata***	nd	nd	Meeßen *et al*. (2013)
*Trebouxia* sp.	ICA	CTT	
***Fulgensia fulgens***	nd	nd	Meeßen *et al*. (2013)
*Trebouxia* sp.	ICA	CTT	
***Lecidea lurida***	nd	nd	Meeßen *et al*. (2013)
*Asterochloris* sp.	nd	CLT, CTT	
***Tephromela atra***	IAA, SA	nd	Pichler *et al*. (2020b)
*Trebouxia* sp.	IAA, ABA, JA, GA_3_, IBA	nd	Pichler *et al*. (2020a)
***Toninia sedifolia***	nd	nd	Meeßen *et al*. (2013)
*Trebouxia* sp.	ICA	CTT	
***Xanthoria elegans***	nd	nd	Meeßen *et al*. (2013)
*Trebouxia* sp.	ICA	CTT	
***Xanthoria parietina***	IAA, SA	nd	Pichler *et al*. (2020b)
*Trebouxia decolorans*	IAA, ABA	nd	Pichler *et al*. (2020a)

			
(b)			
**Mycobiont treatment**	***Cladonia grayi***	***Xanthoria parietina***	***Tephromela atra***
Dim light	0.012 ± 0.012	1.158 ± 0.602	13.526 ± 2.580
			
**Photobiont treatment**	***Asterochloris glomerata***	***Trebouxia decolorans***	***Trebouxia* sp.**
Dim light	0.973 ± 0.155	0.553 ± 0.051	1.290 ± 0.221
High light	1.317 ± 0.311	1.257 ± 0.205	1.280 ± 0.188
DH + dim light	2.927 ± 0.658	1.053 ± 0.407	6.531 ± 1.205
DH + high light	1.371 ± 0.111	0.942 ± 0.389	1.101 ± 0.269

(a) Phytohormones, phytohormone precursors and cyclic peptides released by isolated lichen symbionts. (b) Indole‐3‐acetic acid release rates by isolated lichen photo‐and mycobionts. ABA, abscisic acid; CLT, cyclo‐(l‐leucyl‐l‐tyrosyl); CTT, cyclo‐(l‐tryptophyl‐l‐tryptophyl); ET, ethylene; GA_3_, gibberellin A3; IAA, indole‐3‐acetic acid; ICA, indole‐carbaldehyde; IBA, indole‐3‐butyric acid; JA, jasmonic acid; nd, not determined. Bold and regular text in the left column indicates mycobiont and photobiont species, respectively. (b) Data show extracellular indole‐3‐acetic acid (IAA) release rates in nmol per day (d) by isolated lichen myco‐ and photobionts, normalised to dry mass (nmol IAA g^‐1^ d^‐1^). Values consider the degradation of IAA in the medium according to exponential degradation described by *y* = 100 e^−0.134*x*^. Data were re‐analysed from values in table 1 and fig. 4 of Pichler *et al*. (2020a), and figs 3 and 4 of Pichler *et al*. (2020b). Data are mean ± SD; DH, de‐rehydration cycle.

Studies on phytohormones in fully established lichen thalli, or released by them, provide evidence for the production of ABA, ethylene, gibberellin A3, IAA and zeatin in lichens (Epstein *et al*., 1986; Ott & Schieleit, 1994; Ergün *et al*., 2002), and future studies will most likely show that this short list is rather incomplete. The physiological and metabolic response of lichens to exogenously offered phytohormones shows their relevance to the lichen symbiosis. Ethylene is thought to be involved in lichen response to abiotic stress factors, such as heavy metals (Garty *et al*., 1997; Kauppi *et al*., 1998), and is released by lichen thalli at elevated rates upon hydration (Ott & Schieleit, 1994). Thallus ABA production was enhanced upon rehydration of desiccated thalli (Dietz & Hartung, 1998; Unal *et al*., 2008) and exogenous ABA application improved thallus survival during permanent water saturation (Dietz & Hartung, 1999). In turn, exogenous ABA treatment can enhance the photobiont's desiccation tolerance, as shown for *Peltigera polydactylon* (Beckett *et al*., 2005). In addition, proteins and genes involved in auxin biosynthesis of lichen‐associated bacteria were identified, and bacterial auxins could also influence the growth of the main lichen symbionts (Grube *et al*., 2009; Cernava *et al*., 2017).

In summary, lichen myco‐ and photobionts, and components of the lichen microbiota, can produce various phytohormones, which can be released in significant amounts into the extracellular space, where they are available for perception by other micro‐organisms, potentially affecting their gene expression, metabolism and physiology. The few papers available provide only limited insights, and care must be taken in the interpretation of the data. Future studies should comprise careful identification of the chemical structures of known, or perhaps new, phytohormones, by state‐of‐the‐art hyphenated techniques such as LC–MS/MS, and NMR, in combination with molecular approaches for testing hypotheses, such as using genetically modified myco‐ and photobionts, once sequenced, culturable and amenable to modification. Of the phytohormones studied so far, roles for ethylene in regulating stress response and for ABA in affecting fungal spore germination and hyphal growth seem to emerge. Moreover, ABA and IAA appear to influence lichen water relations, and thus, potentially desiccation tolerance.

## IV. The thallus: a fine‐tuned symbiotic interplay supporting nutrition, tolerance of irradiation and desiccation, and reproduction

### 1. Shunting of macro‐ and micronutrients in the lichen thallus

Whereas at least some knowledge is available regarding the shunting of phosphorous and nitrogen between lichen symbionts, the role of mineral nutrition in the various stages of lichenization still remains a black box that deserves illuminating through future studies. In resynthesized *Usnea hakonensis* thalli (Kono *et al*., 2020), photobiont genes involved in photosynthate transport and mycobiont genes involved in nitrogen and phosphorus transport were upregulated compared with isolated myco‐ and photobionts. Specifically, gene clusters specific for acid phosphatase and phosphate transport were upregulated in the mycobiont, but absent in the photobiont, which upregulated genes for ATPase, arguing for transport of inorganic phosphates from the mycobiont to the photobiont. The upregulation of photosynthate transport reinforces that the photobiont is the main breadwinner providing reduced carbon to the symbiosis, and the mycobiont reciprocates by supplying the photobiont with other elements, such as phosphorous and nitrogen, calcium, magnesium, manganese, copper and iron extracted from the substrate through weathering (Adamo & Violante, 2000), and importantly, water (Honegger, 1991).

Tripartite lichens and cyanolichens benefit from nitrogen fixed by cyanobacteria (Honegger, 1993; Almendras *et al*., 2018), involving significant symbiotic interaction. In a tripartite lichen, *Peltigera aphthosa*, nitrogen fixed by *Nostoc* sp. cyanobacteria was transferred to both, the mycobiont, which incorporated it into amino acids (e.g. alanine, aspartate, glutamine and glutamate), and the *Coccomyxa* photobiont. Over 95% was fixed as NH_4_^+^, supported by cyanobacterial CH_3_NH_3_^+^‐ and NH_4_^+^‐specific transporters and requiring the activity of glutamate dehydrogenase in cyanobacteria (Kershaw & Millbank, 1970; Rai *et al*., 1980, 1983), although further evidence is needed for a better appreciation of the biochemical pathways involved in nitrogen fixation and transport, and their mutual regulation in the tripartite symbiosis. In addition, lichens also host potentially nitrogen‐fixing eubacteria, whose diversity is greater in chlorolichens than in cyanolichens (Almendras *et al*., 2018). Therefore, we suggest that in the differentiation stage, the microbiota could commence supporting the symbiosis, either by directly supplying nutrients to the primary symbionts or indirectly by nutrient release from a decaying microbiota fraction, that is from dead bacteria or fungi.

### 2. Secondary lichen products benefit the established symbiosis

Lichens can produce over 1000 known secondary metabolites, including polyketides such as anthraquinones, chromones, depsides, depsidones, lactones, usnic acid and xanthones. As their occurrence and abundance often correlates well with phylogenetic lineages, they are used as a mainstay in lichen chemosystematics (Calcott *et al*., 2018). Most polyketides are deposited as extracellular crystals on the hyphal cell walls in the upper cortex of the thallus, for example atranorin, parietin and usnic acid, or as granules within hyphae, for example melanins, whereas others accumulate in the medulla, for example physodic acid, physodalic acid, lecanoric acid and protocetraric acid (Solhaug & Gauslaa, 1996; Solhaug *et al*., 2009; Suno *et al*., 2021; Daminova *et al*., 2022). However, it should be noted that not all fungi produce crystallized compounds in the lichenized thallus, notably cyanolichens. Polyketide biosynthetic pathways have not been fully characterized, but genes encoding polyketide synthases constitute one of the largest gene families in fungi, and recent genomic studies reflect the increasing interest in pathway genes generating these compounds (Singh *et al*., 2021a,b; Gerasimova *et al*., 2022). The antimicrobial, allelopathic and antiherbivore properties of many secondary lichen metabolites point to pivotal roles in protecting lichen thalli from biotic stress factors, including in fungus–fungus competition with common moulds, while those with other properties, such as antioxidant and photoprotective properties lend further support to the established symbiosis (Calcott *et al*., 2018; for review). Protection from, and competition with other organisms contributes to shaping community structure and the distribution of individual lichen species (Armstrong & Welch, 2007). For example, lichen substances released into the environment can have allelopathic effects towards other fungi, bacteria, algae, mosses and vascular plants, and also other lichens via influencing photosynthesis, respiration, transpiration, protein and nucleic acid synthesis, membrane ion transport and permeability (Macias *et al*., 2007). Lichen acids such as vulpinic acid, evernic acid and stictic acid exert allelopathic effects within lichen communities, although lichenized fungi have a higher tolerance as compared to non‐lichenized fungi (Whiton & Lawrey, 1984). Usnic acid is toxic to non‐lichen‐forming microalgae such as *Chlamydomonas reinhardii* (Schimmer & Lehner, 1973) and *Scenedesmus quadricauda* (Bačkor *et al*., 2010), and the moss *Physcomitrella patens* (Goga *et al*., 2020). Usnic acid, norstictic acid and parietin are also allelopathic to rock‐inhabiting fungi, other green algae and cyanobacteria (Gazzano *et al*., 2013). Lichen photobionts such as *Trebouxia erici* appear to tolerate usnic acid better, although exogenous application of usnic acid and vulpinic acid can inhibit photobiont growth (Bačkor *et al*., 1998, 2010). Therefore, polyketides produced by lichen mycobionts help sustaining the symbiosis by protection from biotic stress factors, and may also play a role in regulating the number of photobiont cells within a thallus. On the contrary, certain polyketides such as atranorin, usnic acid, and anthraquinones produced by the mycobiont can act as ‘sun‐screen’ pigments in the upper cortex, regulating the amount of incident light received by the photobiont (Solhaug *et al*., 2003; Beckett *et al*., 2021).

Polyketide production by axenically cultured mycobionts differs from that of resynthesizing myco‐ and photobiont co‐cultures (Sveshnikova & Piercey‐Normore, 2021) and does not necessarily correspond to the set of polyketides found in lichen thalli collected in nature. In addition, specific conditions of temperature, light and water availability, and growth media with added sugars or sugar alcohols appear to be required for the production of specific secondary metabolites (Stocker‐Wörgötter, 2002; Elshobary *et al*., 2016). Associated ascomycete or basidiomycete fungi can influence the production of secondary lichen metabolites (Spribille *et al*., 2016; Asplund *et al*., 2018), and in turn, secondary metabolites deposited extracellularly on a lichen can influence bacterial growth, and thus the composition of the lichen microbiota, as summarized by Grimm *et al*. (2021). The mere existence of the lichen microbiota implies that lichen‐associated bacteria have adapted tolerance of lichen compounds. Interestingly, certain gene clusters encoding secondary metabolite synthases are downregulated in *Cladonia rangiferina* co‐cultured with its compatible photobiont, suggesting that potential allelopathic effects towards the photobiont can be avoided in the early stages of lichenization (Sveshnikova & Piercey‐Normore, 2021). Which compounds precisely are affected is not yet clear and considering the large number of gene clusters for biosynthetic pathways present in lichens genomes, linking all biosynthetic genes with products will be a tall order. The differential regulation of these genes is in accordance with the early finding of differential methylation patterns in secondary metabolite genes during lichenization (Armaleo & Miao, 1999). Taken together, the findings discussed in this section suggest that the production of the set of secondary metabolites typically found in a fully formed thallus requires stimuli from the environment, the photobiont and other co‐habitants within the thallus. However, whereas secondary lichen metabolites are key to sustain the fully formed thallus, their downregulation in the early stages of lichenization implies that they are unwanted while establishing first contact. Whether the switch from down‐ to upregulating polyketide synthesis occurs already in the incorporation stage, the differentiation stage or even after establishing a co‐habiting microbiota, is yet to be seen.

### 3. Molecules conferring desiccation tolerance

A key trait of fully established lichen thalli is their remarkable ability to survive desiccation. Desiccation tolerance is often defined as the capability of surviving drying to the air‐dry state at relative humidities below 65%, corresponding to a drop in absolute water content to or below 0.1 g H_2_O g^−1^ dry mass and a water potential of ≤ −100 MPa, and is believed to occur in almost all, if not all, lichens (Kranner *et al*., 2008). Such extremely low water contents can induce ‘vitrification’, that is transition to a ‘glassy state’, of cytoplasmic components dominated by proteins and sugars, causing metabolism to come to a standstill (Kranner *et al*., 2008; Candotto Carniel *et al*., 2021). This state of ‘suspended life’ supports survival under extreme environmental conditions, such as circumpolar habitats that are often dominated by lichens – which is why lichens are often called ‘extremophiles’, as they can thrive under environmental conditions too harsh for vascular plants that rapidly outcompete the slow‐growing lichens under more favourable conditions.

Desiccation is associated with oxidative damage, molecular crowding and Maillard reactions causing damage to macromolecules and subcellular organization, and surviving with almost no water is based on constitutive and inducible protection (Kranner *et al*., 2008; Candotto Carniel *et al*., 2016; Oliver *et al*., 2020). Briefly, known protection mechanisms include an efficient antioxidant system, ‘late embryogenesis abundant’ (LEA)‐like proteins or ‘dehydrins’ and ‘desiccation related proteins’ (DRPs), nonreducing sugars and sugar alcohols (Kranner *et al*., 2005, 2008, 2010; Banchi *et al*., 2018). Together with other ‘natural deep eutectic solvents’ (NADES), these compounds may form ‘functional liquid media’ capable of dissolving molecules of low water solubility (du Toit *et al*., 2021), supporting survival during and after drying. LEA‐like proteins likely act as molecular shields and together with non‐reducing sugars, substitute for water, maintaining the spacing between and within macromolecules (Tunnacliffe & Wise, 2007), avoiding molecular crowding, Maillard reactions and cellular collapse upon desiccation. While a ‘dual role’ for lichen sugar alcohols, being main carbon sources and contributing to desiccation tolerance, has recently been reviewed (Kranner *et al*., 2022; Spribille *et al*., 2022), the involvement of protective proteins in lichen desiccation tolerance has not been investigated, although postulated earlier (Kranner & Lutzoni, 1999), and supported by the finding that constitutively expressed LEA‐like proteins are present in *Trebouxia* photobionts (Candotto Carniel *et al*., 2016). Furthermore, hydrophobins, small cysteine‐rich, hydrophobic proteins on the cell surface of the mycobiont, could play important roles in keeping air‐filled spaces within stratfied foliose and fruticose lichen thalli during water saturation, potentially contributing to oxidative stress avoidance (Honegger, 1993).

Extracellularly deposited lichen polyketides (polyphenols especially) have significant *in vitro* antioxidant activities (Fernández‐Moriano *et al*., 2016). However, survival of desiccation requires scavenging of reactive oxygen species formed upon desiccation by intracellular antioxidants, especially in the light. It is not known whether antioxidants are released extracellularly by free‐living lichen mycobionts and photobionts, but evidence exists that lichenization is accompanied by mutual upregulation of protective systems, shown for the lichen *Cladonia vulcani* (Kranner *et al*., 2005). Without fungal contact, the alga *Trebouxia excentrica* tolerates only very dim light and its carotenoid‐based photoprotection is only partially effective, and without the alga, the glutathione‐based antioxidant system of the fungus is slow and ineffective. However, in the fully formed thallus, antioxidant and photoprotective mechanisms are more effective by orders of magnitude compared with its isolated partners (Kranner *et al*., 2005), vaguely reminiscent of a very different symbiosis, in which photosynthetic sea slugs induce protective changes (such as elevating the plastoquinone pool) to the chloroplasts they steal from algae (Havurinne & Tyystjarvi, 2020). This mutually enhanced resistance to oxidative stress together with enhanced desiccation tolerance supports life above ground in the form of the *Cladonia vulcani* miniature skyscraper, increasing the chance of dispersal of reproductive propagules and ensuring the symbionts' joint evolutionary success (Kranner *et al*., 2005). The ability of prospective lichen symbionts to survive desiccation already before lichenization is a very likely pre‐requisite for them to form a lichen. However, the symbiosis appears to prime the symbionts for becoming even hardier towards the environmental changes, such as elevated irradiance and air dryness, associated with leaving their hidden life below ground, or in shaded habitats such as soil, bark or rock. Moreover, desiccation tolerance requires great cell wall flexibility to support recurrent cellular shrinkage and swelling upon desiccation and rehydration, as shown for isolated lichen photobionts (Gonzalez‐Hourcade *et al*., 2020). Whereas information on the responses of lichens and, less so, on their isolated primary symbionts, to desiccation and rehydration is available, the effects on the lichen microbiota are still to be investigated.

## V. Outlook

**Fig. 6:**
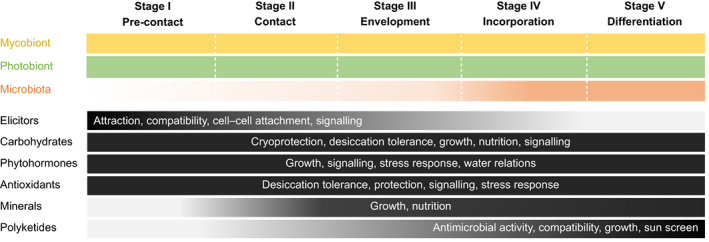
Summary of metabolite groups and signalling molecules proposed to be involved in lichenization. Compounds involved in the five stages of lichenization, none of which are envisaged to be exclusive to a specific phase, are represented in shades from grey to black; darker shades denote a proposed greater contribution to lichenization, also in the bar representing the microbiota. Compounds that elicit contact, such as lectins and cyclic peptides, appear to dominate the early stages, whereas carbon exchange between the symbionts, phytohormone signalling and protection from oxidative damage are suggested to be involved in the lichenization process throughout all stages, whereas polyketides and nutrition with non‐carbon‐based elements such as nitrogen and phosphorous support establishment and maintenance of the thallus, together with the microbiota acquired during and after completion of lichenization.

Many symbioses are formed by including a symbiotic partner in an already established host, or a specific organ of that host, for example roots, symbiosomes and other parts of a macrosymbiont. Lichens differ from these cases, as their thalli arise as a symbiotic structure from the very beginning of a microbial encounter, which requires recognition of the partners and maintenance of the partnership from the earliest stages onwards. Here, we summarized the current knowledge about key metabolites apparently involved in this process. The emerging picture suggests that various metabolite groups work in a concerted manner throughout the various stages of lichenization, with elicitors including lectins and certain cyclic peptides – and surely many other molecules will be found to contribute – supporting contact in the early stages of lichenization. Sugars and sugar alcohols, being the main carbon sources first involved in attracting a prospective mycobiont and later shunted between the symbionts, are very likely pivotal players throughout all stages of lichenization, together with other compound groups, such as secondary lichen products and antioxidants, that contribute to maintaining the fine‐tuned symbiotic interplay within the fully established thallus, and in participation with the lichen microbiota (Fig. 6). This suggests a shifting spectrum of regulatory metabolites from the first encounter of the partners through to stabilizing the fully established lichen thallus. It will be interesting to study, whether and in which way the above‐mentioned metabolite groups contribute to epigenetic regulation of gene expression through DNA methylation, as such a regulation has already been demonstrated in other symbioses (e.g. Satge *et al*., 2016; Li *et al*., 2018). As lichens often grow under austere conditions, it would seem plausible to assume a sustained epigenetic adjustment of symbiotic transcriptomes, a sort of mutual parameter setting, and this may comprise a mutual down‐regulation of defence systems to enable contact. Furthermore, thalli evolved in distantly related fungi, and there are different trajectories to what has convergently evolved in more complex stratified forms. Certainly, much more work lies ahead of us to better understand the process of lichenization from the precontact stage to basal or complex thalli, and the role of metabolites in lichenization. We are convinced that a plethora of further metabolites are yet to be identified, given the tremendous diversity of lichens and the fact that they involve rather different lineages of fungi.

## Competing interests

None declared.

## Data Availability

Data sharing is not applicable to this article as no new data were created or analysed in this study.

## References

[nph18780-bib-0001] Abdulsalam O , Wagner K , Wirth S , Kunert M , David A , Kallenbach M , Boland W , Kothe E , Krause K . 2021. Phytohormones and volatile organic compounds, like geosmin, in the ectomycorrhiza of *Tricholoma vaccinum* and Norway spruce (*Picea abies*). Mycorrhiza 31: 173–188. 33210234 10.1007/s00572-020-01005-2 PMC7910269

[nph18780-bib-0002] Adamo P , Violante P . 2000. Weathering of rocks and neogenesis of minerals associated with lichen activity. Applied Clay Science 16: 229–256.

[nph18780-bib-0003] Ahmadjian V , Jacobs JB . 1981. Relationship between fungus and alga in the lichen *Cladonia cristatella* Tuck. Nature 289: 169–172.

[nph18780-bib-0004] Alen'kina SA , Bogatyrev VA , Matora LY , Sokolova MK , Chernyshova MP , Trutneva KA , Nikitina VE . 2014. Signal effects of the lectin from the associative nitrogen‐fixing bacterium *Azospirillum brasilense* Sp7 in bacterial‐plant root interactions. Plant and Soil 381: 337–349.

[nph18780-bib-0005] Almendras K , García J , Carú M , Orlando J . 2018. Nitrogen‐fixing bacteria associated with *Peltigera* cyanolichens and *Cladonia* chlorolichens. Molecules 23: 3077. 30477264 10.3390/molecules23123077 PMC6320784

[nph18780-bib-0006] Armaleo D , Miao V . 1999. Symbiosis and DNA methylation in the *Cladonia* lichen fungus. Symbiosis 26: 143–163.

[nph18780-bib-0007] Armaleo D , Müller O , Lutzoni F , Andrésson ÓS , Blanc G , Bode HB , Collart FR , Dal Grande F , Dietrich F , Grigoriev IV *et al*. 2019. The lichen symbiosis re‐viewed through the genomes of *Cladonia grayi* and its algal partner *Asterochloris glomerata* . BMC Genomics 20: 605. 31337355 10.1186/s12864-019-5629-x PMC6652019

[nph18780-bib-0008] Armstrong RA , Welch AR . 2007. Competition in lichen communities. Symbiosis 43: 1–12.

[nph18780-bib-0009] Asplund J , Gauslaa Y , Merinero S . 2018. Low synthesis of secondary compounds in the lichen *Lobaria pulmonaria* infected by the lichenicolous fungus *Plectocarpon lichenum* . New Phytologist 217: 1397–1400. 29274278 10.1111/nph.14978

[nph18780-bib-0010] Athukorala SNP , Huebner E , Piercey‐Normore MD . 2014. Identification and comparison of the 3 early stages of resynthesis for the lichen *Cladonia rangiferina* . Canadian Journal of Microbiology 60: 41–52. 24392925 10.1139/cjm-2013-0313

[nph18780-bib-0011] Bačkor M , Hudak J , Repčák M , Ziegler W , Bačkorová M . 1998. The influence of pH and lichen metabolites (vulpinic acid and (+) usnic acid) on the growth of the lichen photobiont *Trebouxia irregularis* . The Lichenologist 30: 577–582.

[nph18780-bib-0012] Bačkor M , Klemová K , Bačkorová M , Ivanova V . 2010. Comparison of the phytotoxic effects of usnic acid on cultures of free‐living alga *Scenedesmus quadricauda* and aposymbiotically grown lichen photobiont *Trebouxia erici* . Journal of Chemical Ecology 36: 405–411. 20306219 10.1007/s10886-010-9776-4

[nph18780-bib-0013] Banchi E , Candotto Carniel F , Montagner A , Petruzzellis F , Pichler G , Giarola V , Bartels D , Pallavicini A , Tretiach M . 2018. Relation between water status and desiccation‐affected genes in the lichen photobiont *Trebouxia gelatinosa* . Plant Physiology and Biochemistry 129: 189–197. 29894859 10.1016/j.plaphy.2018.06.004

[nph18780-bib-0014] Bandurski RS , Cohen JD , Slovin JP , Reinecke DM . 1995. Auxin biosynthesis and metabolism. In: Davies PJ , ed. Plant hormones: physiology, biochemistry and molecular biology. Dordrecht, the Netherlands: Springer, 39–65.

[nph18780-bib-0015] Basso V , Kohler A , Miyauchi S , Singan V , Guinet F , Šimura J , Novák O , Barry KW , Amirebrahimi M , Block J *et al*. 2020. An ectomycorrhizal fungus alters sensitivity to jasmonate, salicylate, gibberellin, and ethylene in host roots. Plant, Cell & Environment 43: 1047–1068. 10.1111/pce.13702 31834634

[nph18780-bib-0016] Becker B . 2013. Snow ball earth and the split of Streptophyta and Chlorophyta. Trends in Plant Science 18: 180–183. 23102566 10.1016/j.tplants.2012.09.010

[nph18780-bib-0017] Beckett RP , Mayaba N , Minibayeva FV , Alyabyev AJ . 2005. Hardening by partial dehydration and ABA increase desiccation tolerance in the cyanobacterial lichen *Peltigera polydactylon* . Annals of Botany 96: 109–115. 15857849 10.1093/aob/mci156 PMC4246815

[nph18780-bib-0018] Beckett RP , Minibayeva F , Solhaug KA , Roach T . 2021. Photoprotection in lichens: adaptations of photobionts to high light. The Lichenologist 53: 21–33.

[nph18780-bib-0019] Bornet E . 1872. Sur les gonidies des lichens. Comptes Rendus 74: 820.

[nph18780-bib-0020] Brunauer G , Hager A , Grube M , Türk R , Stocker‐Worgotter E . 2007. Alterations in secondary metabolism of aposymbiotically grown mycobionts of *Xanthoria elegans* and cultured resynthesis stages. Plant Physiology and Biochemistry 45: 146–151. 17344057 10.1016/j.plaphy.2007.01.004

[nph18780-bib-0021] Bubrick P , Galun M . 1980. Proteins from the lichen *Xanthoria parietina* which bind to phycobiont cell walls. Correlation between binding patterns and cell wall cytochemistry. Protoplasma 104: 167–173.

[nph18780-bib-0022] Bubrick P , Galun M , Frensdorff A . 1984. Observations on free‐living *Trebouxia* de Puymaly and *Pseudotrebouxia* Archibald, and evidence that both symbionts from *Xanthoria parietina* (L.) TH. FR. can be found free‐living in nature. New Phytologist 97: 455–462.

[nph18780-bib-0023] Calcott MJ , Ackerley DF , Knight A , Keyzers RA , Owen JG . 2018. Secondary metabolism in the lichen symbiosis. Chemical Society Reviews 47: 1730–1760. 29094129 10.1039/c7cs00431a

[nph18780-bib-0024] Candotto Carniel F , Fernandez‐Marín B , Arc E , Craighero T , Laza JM , Incerti G , Tretiach M , Kranner I . 2021. How dry is dry? Molecular mobility in relation to thallus water content in a lichen. Journal of Experimental Botany 72: 1576–1588. 33165603 10.1093/jxb/eraa521

[nph18780-bib-0025] Candotto Carniel F , Gerdol M , Montagner A , Banchi E , De Moro G , Manfrin C , Muggia L , Pallavicini A , Tretiach M . 2016. New features of desiccation tolerance in the lichen photobiont *Trebouxia gelatinosa* are revealed by a transcriptomic approach. Plant Molecular Biology 91: 319–339. 26992400 10.1007/s11103-016-0468-5

[nph18780-bib-0026] Cernava T , Erlacher A , Aschenbrenner IA , Krug L , Lassek C , Riedel K , Grube M , Berg G . 2017. Deciphering functional diversification within the lichen microbiota by meta‐omics. Microbiome 5: 82. 28724401 10.1186/s40168-017-0303-5 PMC5518139

[nph18780-bib-0027] Chanclud E , Morel JB . 2016. Plant hormones: a fungal point of view. Molecular Plant Pathology 17: 1289–1297. 26950404 10.1111/mpp.12393 PMC6638337

[nph18780-bib-0028] Costacurta A , Vanderleyden J . 1995. Synthesis of phytohormones by plant‐associated bacteria. Critical Reviews in Microbiology 21: 1–18. 7576148 10.3109/10408419509113531

[nph18780-bib-0029] Daminova AG , Rogov AM , Rassabina AE , Beckett RP , Minibayeva FV . 2022. Effect of melanization on thallus microstructure in the lichen *Lobaria pulmonaria* . Journal of Fungi 8: 791. 36012780 10.3390/jof8080791 PMC9409904

[nph18780-bib-0030] De Los RA , Ascaso C , Grube M . 2002. An ultrastructural, anatomical and molecular study of the lichenicolous lichen *Rimularia insularis* . Mycological Research 106: 946–953.

[nph18780-bib-0031] Decker EL , Frank W , Sarnighausen E , Reski R . 2006. Moss systems biology en route: phytohormones in *Physcomitrella* development. Plant Biology 8: 397–405. 16807833 10.1055/s-2006-923952

[nph18780-bib-0032] Delaux PM , Radhakrishnan GV , Jayaraman D , Cheem J , Malbreil M , Volkening JD , Sekimoto H , Nishiyama T , Melkonian M , Pokorny L *et al*. 2015. Algal ancestor of land plants was preadapted for symbiosis. Proceedings of the National Academy of Sciences, USA 112: 13390–13395. 10.1073/pnas.1515426112 PMC4629359 26438870

[nph18780-bib-0033] Díaz EM , Sánchez‐Elordi E , Santiago R , Vicente C , Legaz ME . 2016. Algal‐fungal mutualism: cell recognition and maintenance of the symbiotic status of lichens. Journal of Veterinary Medicine and Research 3: 1052.

[nph18780-bib-0034] Díaz EM , Vicente‐Manzanares M , Sacristán M , Vicente C , Legaz ME . 2011. Fungal lectin of *Peltigera canina* induces chemotropism of compatible *Nostoc* cells by constriction‐relaxation pulses of cyanobiont cytoskeleton. Plant Signaling & Behavior 6: 1525–1536. 21897128 10.4161/psb.6.10.16687 PMC3256381

[nph18780-bib-0035] Dietz S , Hartung W . 1998. Abscisic acid in lichens: variation, water relations and metabolism. New Phytologist 138: 99–106.

[nph18780-bib-0036] Dietz S , Hartung W . 1999. The effect of abscisic acid on chlorophyll fluorescence in lichens under extreme water regimes. New Phytologist 143: 495–501. 33862889 10.1046/j.1469-8137.1999.00473.x

[nph18780-bib-0037] Douglas AE . 2014. The symbiotic habit. Princeton, NJ, USA: Princeton University Press.

[nph18780-bib-0038] Drew EA , Smith DC . 1967. Studies in the physiology of lichens. VII. The physiology of the *Nostoc* symbiont of *Peltigera polydactyla* compared with cultured and free‐living forms. New Phytologist 66: 379–388.

[nph18780-bib-0039] Eisenreich W , Knispel N , Beck A . 2011. Advanced methods for the study of the chemistry and the metabolism of lichens. Phytochemistry Reviews 10: 445–456.

[nph18780-bib-0040] Ek M , Ljungquist PO , Stenström E . 1983. Indole‐3‐acetic acid production by mycorrhizal fungi determined by gas chromatography‐mass spectrometry. New Phytologist 94: 401–407.

[nph18780-bib-0041] Elshobary ME , Osman ME , Abo‐Shady AM , Komatsu E , Perreault H , Sorensen J , Piercey‐Normore MD . 2016. Algal carbohydrates affect polyketide synthesis of the lichen‐forming fungus *Cladonia rangiferina* . Mycologia 108: 646–656. 27091386 10.3852/15-263

[nph18780-bib-0042] Epstein E , Sagee O , Cohen JD , Garty J . 1986. Endogenous auxin and ethylene in the lichen *Ramalina duriaei* . Plant Physiology 82: 1122–1125. 16665145 10.1104/pp.82.4.1122 PMC1056269

[nph18780-bib-0043] Ergün N , Topcuoğlu ŞF , Yildiz A . 2002. Auxin (ndole‐3‐acetic acid), gibberellic acid (GA_3_), abscisic acid (ABA) and cytokinin (zeatin) production by some species of mosses and lichens. Turkish Journal of Botany 26: 13–18.

[nph18780-bib-0044] Erlacher A , Cernava T , Cardinale M , Soh J , Sensen CW , Grube M , Berg G . 2015. Rhizobiales as functional and endosymbiontic members in the lichen symbiosis of *Lobaria pulmonaria* L. Frontiers in Microbiology 6: 53. 25713563 10.3389/fmicb.2015.00053 PMC4322706

[nph18780-bib-0045] Fernández‐Moriano C , Gómez‐Serranillos MP , Crespo A . 2016. Antioxidant potential of lichen species and their secondary metabolites. A systematic review. Pharmaceutical Biology 54: 1–17. 25885942 10.3109/13880209.2014.1003354

[nph18780-bib-0046] Fontaniella B , Vicente C , Legaz ME . 2000. The cryoprotective role of polyols in lichens: effects on the redistribution of RNase in *Evernia prunastri* thallus during freezing. Plant Physiology and Biochemistry 38: 621–627.

[nph18780-bib-0047] Foo E , Plett JM , Lopez‐Raez JA , Reid D . 2019. Editorial: the role of plant hormones in plant‐microbe symbioses. Frontiers in Plant Science 10: 1391. 31737016 10.3389/fpls.2019.01391 PMC6832933

[nph18780-bib-0048] Galun M , Braun A , Frensdorff A , Galun E . 1976. Hyphal walls of isolated lichen fungi ‐ Autoradiographic localization of precursor incorporation and binding of fluorescein‐conjugated lectins. Archives of Microbiology 108: 9–16. 1275648 10.1007/BF00425087

[nph18780-bib-0049] Garty J , Kloog N , Wolfson R , Cohen Y , Karnieli A , Avni A . 1997. The influence of air pollution on the concentration of mineral elements, on the spectral reflectance response and on the production of stress‐ethylene in the lichen *Ramalina duriaei* . New Phytologist 137: 587–597.

[nph18780-bib-0050] Gay G , Normand L , Marmeisse R , Sotta B , Debaud JC . 1994. Auxin overproducer mutants of *Hebeloma cylindrosporum* Romagnesi have increased mycorrhizal activity. New Phytologist 128: 645–657.

[nph18780-bib-0051] Gazarian IG , Lagrimini LM , Mellon FA , Naldrett MJ , Ashby GA , Thorneley RNF . 1998. Identification of skatolyl hydroperoxide and its role in the peroxidase‐catalysed oxidation of indol‐3‐yl acetic acid. Biochemical Journal 333: 223–232. 9639583 10.1042/bj3330223 PMC1219576

[nph18780-bib-0052] Gazzano C , Favero‐Longo SE , Iacomussi P , Piervittori R . 2013. Biocidal effect of lichen secondary metabolites against rock‐dwelling microcolonial fungi, cyanobacteria and green algae. International Biodeterioration & Biodegradation 84: 300–306.

[nph18780-bib-0053] Gerasimova JV , Beck A , Werth S , Resl P . 2022. High diversity of type I polyketide genes in *Bacidia rubella* as revealed by the comparative analysis of 23 lichen genomes. Journal of Fungi 8: 449. 35628705 10.3390/jof8050449 PMC9146135

[nph18780-bib-0054] Goga M , Elečko J , Marcinčinová M , Ručová D , Bačkorová M , Bačkor M . 2020. Lichen metabolites: an overview of some secondary metabolites and their biological potential. In: Mérillon J‐M , Ramawat KG , eds. Co‐evolution of secondary metabolites. Cham, Switzerland: Springer International Publishing, 175–209.

[nph18780-bib-0055] Gonzalez‐Hourcade M , Braga MR , del Campo EM , Ascaso C , Patino C , Casano LM . 2020. Ultrastructural and biochemical analyses reveal cell wall remodelling in lichen‐forming microalgae submitted to cyclic desiccation‐rehydration. Annals of Botany 125: 459–469. 31679006 10.1093/aob/mcz181 PMC7061176

[nph18780-bib-0056] Grimm M , Grube M , Schiefelbein U , Zühlke D , Bernhardt J , Riedel K . 2021. The lichens' microbiota, still a mystery? Frontiers in Microbiology 12: 623839. 33859626 10.3389/fmicb.2021.623839 PMC8042158

[nph18780-bib-0057] Grube M , Berg G . 2009. Microbial consortia of bacteria and fungi with focus on the lichen symbiosis. Fungal Biology Reviews 23: 72–85.

[nph18780-bib-0058] Grube M , Cardinale M , de Castro JV , Müller H , Berg G . 2009. Species‐specific structural and functional diversity of bacterial communities in lichen symbioses. ISME Journal 3: 1105–1115. 19554038 10.1038/ismej.2009.63

[nph18780-bib-0059] Grzesiak J , Woltyńska A , Zdanowski MK , Górniak D , Świątecki A , Olech MA , Aleksandrzak‐Piekarczyk T . 2021. Metabolic fingerprinting of the Antarctic cyanolichen *Leptogium puberulum*‐associated bacterial community (Western Shore of Admiralty Bay, King George Island, Maritime Antarctica). Microbial Ecology 82: 818–829. 33555368 10.1007/s00248-021-01701-2 PMC8674174

[nph18780-bib-0060] Hájek J , Váczi P , Barták M . 2009a. Photosynthetic electron transport at low temperatures in the green algal foliose lichens *Lasallia pustulata* and *Umbilicaria hirsuta* affected by manipulated levels of ribitol. Photosynthetica 47: 199–205.

[nph18780-bib-0061] Hájek J , Váczi P , Barták M , Smejkal L , Lipavská H . 2009b. Cryoproective role of ribitol in *Xanthoparmelia somloensis* . Biologia Plantarum 53: 677–684.

[nph18780-bib-0062] Hametner C , Stocker‐Wörgötter E , Grube M . 2014. New insights into diversity and selectivity of trentepohlialean lichen photobionts from the extratropics. Symbiosis 63: 31–40. 25076805 10.1007/s13199-014-0285-z PMC4110408

[nph18780-bib-0063] Havurinne V , Tyystjarvi E . 2020. Photosynthetic sea slugs induce protective changes to the light reactions of the chloroplasts they steal from algae. eLife 9: 29. 10.7554/eLife.57389 PMC7679141 33077025

[nph18780-bib-0064] Hawksworth DL , Grube M . 2020. Lichens redefined as complex ecosystems. New Phytologist 227: 1281–1283. 32484275 10.1111/nph.16630 PMC7497170

[nph18780-bib-0065] Hill DJ . 1989. The control of the cell cycle in microbial symbionts. New Phytologist 112: 175–184.

[nph18780-bib-0066] Hill DJ . 1993. The co‐ordination of development of symbionts in mutualistic symbiosis with reference to the cell cycle of the photobiont in lichens. Symbiosis 14: 325–333.

[nph18780-bib-0067] Hill DJ , Smith DC . 1972. Lichen physiology. XII. 'The inhibition technique'. New Phytologist 71: 15–30.

[nph18780-bib-0068] Honegger R . 1986. Ultrastructural studies in lichens. I. Haustorial types and their frequencies in a range of lichens with trebouxioid photobionts. New Phytologist 103: 785–795.

[nph18780-bib-0069] Honegger R . 1991. Functional aspects of the lichen symbiosis. Annual Review of Plant Physiology and Plant Molecular Biology 42: 553–578.

[nph18780-bib-0070] Honegger R . 1993. Developmental biology of lichens. New Phytologist 125: 659–677. 33874446 10.1111/j.1469-8137.1993.tb03916.x

[nph18780-bib-0071] Honegger R . 2012. 15 The symbiotic phenotype of lichen‐forming ascomycetes and their endo‐ and epibionts. In: Hock B , ed. Fungal associations. Berlin, Heidelberg, Germany: Springer, 287–339.

[nph18780-bib-0072] Honegger R , Edwards D , Axe L . 2013. The earliest records of internally stratified cyanobacterial and algal lichens from the lower devonian of the Welsh borderland. New Phytologist 197: 264–275. 23110612 10.1111/nph.12009

[nph18780-bib-0073] Honegger R , Kutasi V , Ruffner HP . 1993. Polyol patterns in eleven species of aposymbiotically cultured lichen mycobionts. Mycological Research 97: 35–39.

[nph18780-bib-0074] Insarova ID , Blagoveshchenskaya EY . 2016. Lichen symbiosis: search and recognition of partners. Biology Bulletin 43: 408–418. 30226935

[nph18780-bib-0075] Joneson S , Armaleo D , Lutzoni F . 2011. Fungal and algal gene expression in early developmental stages of lichen‐symbiosis. Mycologia 103: 291–306. 20943535 10.3852/10-064

[nph18780-bib-0076] Joneson S , Lutzoni F . 2009. Compatibility and thigmotropism in the lichen symbiosis: a reappraisal. Symbiosis 47: 109–115.

[nph18780-bib-0077] Kauppi M , Kauppi A , Garty J . 1998. Ethylene produced by the lichen *Cladina stellaris* exposed to sulphur and heavy‐metal‐containing solutions under acidic conditions. New Phytologist 139: 537–547.

[nph18780-bib-0078] Kershaw KA , Millbank JW . 1970. Nitrogen metabolism in lichens II. The partition of cephalodial fixed nitrogen between the mycobiont and phycobionts of *Peltigera aphthosa* . New Phytologist 69: 75–79.

[nph18780-bib-0079] Komiya T , Shibata S . 1971. Polyols produced by the cultured phyco‐ and mycobionts of some *Ramalina* species. Phytochemistry 10: 695–699.

[nph18780-bib-0080] Kono M , Kon Y , Ohmura Y , Satta Y , Terai Y . 2020. In vitro resynthesis of lichenization reveals the genetic background of symbiosis‐specific fungal‐algal interaction in *Usnea hakonensis* . BMC Genomics 21: 671. 32993496 10.1186/s12864-020-07086-9 PMC7526373

[nph18780-bib-0081] Kono M , Tanabe H , Ohmura Y , Satta Y , Terai Y . 2017. Physical contact and carbon transfer between a lichen‐forming *Trebouxia* alga and a novel Alphaproteobacterium. Microbiology 163: 678–691. 28535846 10.1099/mic.0.000461

[nph18780-bib-0082] Kosugi M , Miyake H , Yamakawa H , Shibata Y , Miyazawa A , Sugimura T , Satoh K , Itoh S , Kashino Y . 2013. Arabitol provided by lichenous fungi enhances ability to dissipate excess light energy in a symbiotic green alga under desiccation. Plant and Cell Physiology 54: 1316–1325. 23737501 10.1093/pcp/pct079

[nph18780-bib-0083] Kranner I , Beckett R , Hochman A , Nash TH . 2008. Desiccation‐tolerance in lichens: a review. Bryologist 111: 576–593.

[nph18780-bib-0084] Kranner I , Cram WJ , Zorn M , Wornik S , Yoshimura I , Stabentheiner E , Pfeifhofer HW . 2005. Antioxidants and photoprotection in a lichen as compared with its isolated symbiotic partners. Proceedings of the National Academy of Sciences, USA 102: 3141–3146. 10.1073/pnas.0407716102 PMC549463 15710882

[nph18780-bib-0085] Kranner I , Lutzoni F . 1999. Evolutionary consequences of transition to a lichen symbiotic state and physiological adaptation to oxidative damage associated with poikilohydry. In: Lerner HR , ed. Plant responses to environmental stresses: from phytohormones to genome reorganization. New York, NY, USA: Routledge, 38.

[nph18780-bib-0086] Kranner I , Minibayeva FV , Beckett RP , Seal CE . 2010. What is stress? Concepts, definitions and applications in seed science. New Phytologist 188: 655–673. 20854396 10.1111/j.1469-8137.2010.03461.x

[nph18780-bib-0087] Kranner I , Pichler G , Grube M . 2022. The lichen market place. New Phytologist 234: 1541–1543. 35478327 10.1111/nph.18130 PMC9321073

[nph18780-bib-0088] Lawrey JD , Diederich P . 2003. Lichenicolous fungi: interactions, evolution, and biodiversity. Bryologist 106: 80–120.

[nph18780-bib-0089] Legaz ME , Fontaniella B , Millanes AM , Vicente C . 2004. Secreted arginases from phylogenetically far‐related lichen species act as cross‐recognition factors for two different algal cells. European Journal of Cell Biology 83: 435–446. 15506567 10.1078/0171-9335-00384

[nph18780-bib-0090] Li Y , Liew YJ , Cui G , Cziesielski MJ , Zahran N , Michell CT , Voolstra CR , Aranda M . 2018. DNA methylation regulates transcriptional homeostasis of algal endosymbiosis in the coral model *Aiptasia* . Science Advances 4: eaat2142. 30116782 10.1126/sciadv.aat2142 PMC6093633

[nph18780-bib-0091] Liao DH , Wang SS , Cui MM , Liu JH , Chen AQ , Xu GH . 2018. Phytohormones regulate the development of arbuscular mycorrhizal symbiosis. International Journal of Molecular Sciences 19: 3146. 30322086 10.3390/ijms19103146 PMC6213213

[nph18780-bib-0092] Lines CEM , Ratcliffe RG , Rees TAV , Southon TE . 1989. A ^13^C NMR study of photosynthate transport and metabolism in the lichen *Xanthoria calcicola* Oxner. New Phytologist 111: 447–456. 33874007 10.1111/j.1469-8137.1989.tb00707.x

[nph18780-bib-0093] Lücking R , Hodkinson BP , Leavitt SD . 2016. The 2016 classification of lichenized fungi in the Ascomycota and Basidiomycota: approaching one thousand genera. Bryologist 119: 361–416.

[nph18780-bib-0094] Lutzoni F , Pagel M , Reeb V . 2001. Major fungal lineages are derived from lichen symbiotic ancestors. Nature 411: 937–940. 11418855 10.1038/35082053

[nph18780-bib-0095] Macias FA , Molinillo JMG , Varela RM , Galindo JCG . 2007. Allelopathy – a natural alternative for weed control. Pest Management Science 63: 327–348. 17348068 10.1002/ps.1342

[nph18780-bib-0096] Meeßen J , Eppenstein S , Ott S . 2013. Recognition mechanisms during the pre‐contact state of lichens: II. Influence of algal exudates and ribitol on the response of the mycobiont of *Fulgensia bracteata* . Symbiosis 59: 131–143.

[nph18780-bib-0097] Meeßen J , Ott S . 2013. Recognition mechanisms during the pre‐contact state of lichens: I. Mycobiont‐photobiont interactions of the mycobiont of *Fulgensia bracteata* . Symbiosis 59: 121–130.

[nph18780-bib-0098] Molina MC , Vicente C . 1995. Correlationships between enzymatic activity of lectins, putrescine content and chloroplast damage in *Xanthoria parietina* phycobionts. Cell Adhesion and Communication 3: 1–12. 7749719 10.3109/15419069509081274

[nph18780-bib-0099] Muggia L , Baloch E , Stabentheiner E , Grube M , Wedin M . 2011. Photobiont association and genetic diversity of the optionally lichenized fungus *Schizoxylon albescens* . FEMS Microbiology Ecology 75: 255–272. 21133956 10.1111/j.1574-6941.2010.01002.x

[nph18780-bib-0100] Muggia L , Fleischhacker A , Kopun T , Grube M . 2016. Extremotolerant fungi from alpine rock lichens and their phylogenetic relationships. Fungal Diversity 76: 119–142. 26877720 10.1007/s13225-015-0343-8 PMC4739527

[nph18780-bib-0101] Nelsen MP , Lücking R , Boyce CK , Lumbsch HT , Ree RH . 2020. No support for the emergence of lichens prior to the evolution of vascular plants. Geobiology 18: 3–13. 31729136 10.1111/gbi.12369

[nph18780-bib-0102] Oliver MJ , Farrant JM , Hilhorst HWM , Mundree S , Williams B , Bewley JD . 2020. Desiccation tolerance: avoiding cellular damage during drying and rehydration. In: Merchant SS , ed. Annual Review of Plant Biology, vol. 71. Palo Alto, CA, USA: Annual Reviews, 435–460. 10.1146/annurev-arplant-071219-105542 32040342

[nph18780-bib-0103] Ott S , Schieleit P . 1994. Influence of exogenous factors on ethylene production by lichens. I. Influence of water content and water status conditions on ethylene production. Symbiosis 16: 187–201.

[nph18780-bib-0104] Parniske M . 2008. Arbuscular mycorrhiza: the mother of plant root endosymbioses. Nature Reviews Microbiology 6: 763–775. 18794914 10.1038/nrmicro1987

[nph18780-bib-0105] Peksa O , Gebouská T , Škvorová Z , Vančurová L , Škaloud P . 2022. The guilds in green algal lichens‐an insight into the life of terrestrial symbiotic communities. FEMS Microbiology Ecology 98: 1–17. 10.1093/femsec/fiac008 35134923

[nph18780-bib-0106] Pichler G , Carniel FC , Muggia L , Holzinger A , Tretiach M , Kranner I . 2021. Enhanced culturing techniques for the mycobiont isolated from the lichen *Xanthoria parietina* . Mycological Progress 20: 797–808. 34720793 10.1007/s11557-021-01707-7 PMC8550697

[nph18780-bib-0107] Pichler G , Stöggl W , Candotto Carniel F , Muggia L , Ametrano CG , Holzinger A , Tretiach M , Kranner I . 2020a. Abundance and extracellular release of phytohormones in aero‐terrestrial microalgae (Trebouxiophyceae, Chlorophyta) as a potential chemical signaling source. Journal of Phycology 56: 1295–1307. 32452544 10.1111/jpy.13032 PMC7689701

[nph18780-bib-0108] Pichler G , Stöggl W , Trippel D , Candotto Carniel F , Muggia L , Ametrano CG , Cimen T , Holzinger A , Tretiach M , Kranner I . 2020b. Phytohormone release by three isolated lichen mycobionts and the effects of indole‐3‐acetic acid on their compatible photobionts. Symbiosis 82: 95–108. 33223597 10.1007/s13199-020-00721-9 PMC7671983

[nph18780-bib-0109] Pozo MJ , Azcón‐Aguilar C . 2007. Unraveling mycorrhiza‐induced resistance. Current Opinion in Plant Biology 10: 393–398. 17658291 10.1016/j.pbi.2007.05.004

[nph18780-bib-0110] Pozo MJ , López‐Ráez JA , Azcón‐Aguilar C , García‐Garrido JM . 2015. Phytohormones as integrators of environmental signals in the regulation of mycorrhizal symbioses. New Phytologist 205: 1431–1436. 25580981 10.1111/nph.13252

[nph18780-bib-0111] Rai AN , Rowell P , Stewart WDP . 1980. NH_4_ ^+^ assimilation and nitrogenase regulation in the lichen *Peltigera aphthosa* Willd. New Phytologist 85: 545–555.

[nph18780-bib-0112] Rai AN , Rowell P , Stewart WDP . 1983. Interactions between cyanobacterium and fungus during ^15^N_2_‐incorporation and metabolism in the lichen *Peltigera canina* . Archives of Microbiology 134: 136–142.

[nph18780-bib-0113] Resl P , Bujold AR , Tagirdzhanova G , Meidl P , Rallo SF , Kono M , Fernández‐Brime S , Guðmundsson H , Andrésson OS , Muggia L *et al*. 2022. Large differences in carbohydrate degradation and transport potential among lichen fungal symbionts. Nature Communications 13: 2634. 10.1038/s41467-022-30218-6 PMC9098629 35551185

[nph18780-bib-0114] Richardson DH , Hill DJ , Smith DC . 1968. Lichen physiology: XI. The role of the alga in determining the pattern of carbohydrate movement between lichen symbionts. New Phytologist 67: 469–486.

[nph18780-bib-0115] Romero JAF , Paglini MG , Priano C , Koroch A , Rodríguez Y , Sailer J , Teleshova N . 2021. Algal and cyanobacterial lectins and their antimicrobial properties. Marine Drugs 19: 687. 34940686 10.3390/md19120687 PMC8707200

[nph18780-bib-0116] Sacristán M , Millanes AM , Legaz ME , Vicente C . 2006. A lichen lectin specifically binds to the α‐1,4‐polygalactoside moiety of urease located in the cell wall of homologous algae. Plant Signaling & Behavior 1: 23–27. 19521472 10.4161/psb.1.1.2276 PMC2633696

[nph18780-bib-0117] Sanders WB . 2002. In situ development of the foliicolous lichen *Phyllophiale* (Trichotheliaceae) from propagule germination to propagule production. American Journal of Botany 89: 1741–1746. 21665600 10.3732/ajb.89.11.1741

[nph18780-bib-0118] Sanders WB , Lücking R . 2002. Reproductive strategies, relichenization and thallus development observed *in situ* in leaf‐dwelling lichen communities. New Phytologist 155: 425–435. 33873320 10.1046/j.1469-8137.2002.00472.x

[nph18780-bib-0119] Santner A , Calderon‐Villalobos LIA , Estelle M . 2009. Plant hormones are versatile chemical regulators of plant growth. Nature Chemical Biology 5: 301–307. 19377456 10.1038/nchembio.165

[nph18780-bib-0120] Satge C , Moreau S , Sallet E , Lefort G , Auriac MC , Rembliere C , Cottret L , Gallardo K , Noirot C , Jardinaud MF *et al*. 2016. Reprogramming of DNA methylation is critical for nodule development in *Medicago truncatula* . Nature Plants 2: 16166. 27797357 10.1038/nplants.2016.166

[nph18780-bib-0121] Schieleit P , Ott S . 1996. Ethylene production and 1‐aminocyclopropane‐1‐carboxylic acid content of lichen bionts. Symbiosis 21: 223–231.

[nph18780-bib-0122] Schimmer O , Lehner H . 1973. Untersuchungen zur Wirkung von Usninsäure auf die Grünalge *Chlamydomonas reinhardii* . Archiv für Mikrobiologie 93: 145–154. 4764231

[nph18780-bib-0123] Schwendener S . 1869. Die Algentypen der Flechtengonidien: Programm für Rectoratsfeier der Universität. Basel, Switzerland: Universitätsbuchdruckerei von C. Schultze.

[nph18780-bib-0124] Selosse MA , Le Tacon F . 1998. The land flora: a phototroph‐fungus partnership? Trends in Ecology & Evolution 13: 15–20. 21238179 10.1016/s0169-5347(97)01230-5

[nph18780-bib-0125] Selosse MA , Strullu‐Derrien C , Martin FM , Kamoun S , Kenrick P . 2015. Plants, fungi and oomycetes: a 400‐million year affair that shapes the biosphere. New Phytologist 206: 501–506. 25800616 10.1111/nph.13371

[nph18780-bib-0126] Singh G , Armaleo D , Dal Grande F , Schmitt I . 2021a. Depside and depsidone synthesis in lichenized fungi comes into focus through a genome‐wide comparison of the olivetoric acid and physodic acid chemotypes of *Pseudevernia furfuracea* . Biomolecules 11: 1445. 34680078 10.3390/biom11101445 PMC8533459

[nph18780-bib-0127] Singh G , Calchera A , Schulz M , Drechsler M , Bode HB , Schmitt I , Dal Grande F . 2021b. Climate‐specific biosynthetic gene clusters in populations of a lichen‐forming fungus. Environmental Microbiology 23: 4260–4275. 34097344 10.1111/1462-2920.15605

[nph18780-bib-0128] Singh RS , Walia AK . 2014. Characteristics of lichen lectins and their role in symbiosis. Symbiosis 62: 123–134.

[nph18780-bib-0129] Solhaug KA , Gauslaa Y . 1996. Parietin, a photoprotective secondary product of the lichen *Xanthoria parietina* . Oecologia 108: 412–418. 28307855 10.1007/BF00333715

[nph18780-bib-0130] Solhaug KA , Gauslaa Y , Nybakken L , Bilger W . 2003. UV‐induction of sun‐screening pigments in lichens. New Phytologist 158: 91–100.

[nph18780-bib-0131] Solhaug KA , Lind M , Nybakken L , Gauslaa Y . 2009. Possible functional roles of cortical depsides and medullary depsidones in the foliose lichen *Hypogymnia physodes* . Flora – Morphology, Distribution, Functional Ecology of Plants 204: 40–48.

[nph18780-bib-0132] Spribille T . 2018. Relative symbiont input and the lichen symbiotic outcome. Current Opinion in Plant Biology 44: 57–63. 29529531 10.1016/j.pbi.2018.02.007

[nph18780-bib-0133] Spribille T , Resl P , Stanton DE , Tagirdzhanova G . 2022. Evolutionary biology of lichen symbioses. New Phytologist 234: 1566–1582. 35302240 10.1111/nph.18048

[nph18780-bib-0134] Spribille T , Tuovinen V , Resl P , Vanderpool D , Wolinski H , Aime MC , Schneider K , Stabentheiner E , Toome‐Heller M , Thor G *et al*. 2016. Basidiomycete yeasts in the cortex of ascomycete macrolichens. Science 353: 488–492. 27445309 10.1126/science.aaf8287 PMC5793994

[nph18780-bib-0135] Stocker‐Wörgötter E . 2001a. Experimental studies of the lichen symbiosis: DNA‐analyses, differentiation and secondary chemistry of selected mycobionts, artificial resynthesis of two‐ and tripartite symbioses. Symbiosis 30: 207–227.

[nph18780-bib-0136] Stocker‐Wörgötter E . 2001b. New frontiers in bryology and lichenology: experimental lichenology and microbiology of lichens: culture experiments, secondary chemistry of cultured mycobionts, resynthesis, and thallus morphogenesis. Bryologist 104: 576–581.

[nph18780-bib-0137] Stocker‐Wörgötter E . 2002. Investigating the production of secondary compounds in cultured lichen mycobionts. In: Kranner IC , Beckett RP , Varma AK , eds. Protocols in lichenology: culturing, biochemistry, ecophysiology and use in biomonitoring. Berlin, Heidelberg, Germany: Springer, 296–306.

[nph18780-bib-0138] Stocker‐Wörgötter E , Türk R . 1991. Artificial resynthesis of thalli of the cyanobacterial lichen *Peltigera praetextata* under laboratory conditions. The Lichenologist 23: 127–138.

[nph18780-bib-0139] Suno H , Machida M , Dohi T , Ohmura Y . 2021. Quantum chemical calculation studies toward microscopic understanding of retention mechanism of Cs radioisotopes and other alkali metals in lichens. Scientific Reports 11: 8228. 33859257 10.1038/s41598-021-87617-w PMC8050294

[nph18780-bib-0140] Sveshnikova N , Piercey‐Normore MD . 2021. Transcriptome comparison of secondary metabolite biosynthesis genes expressed in cultured and lichenized conditions of *Cladonia rangiferina* . Diversity‐Basel 13: 529.

[nph18780-bib-0141] Tagirdzhanova G , Saary P , Tingley JP , Díaz‐Escandón D , Abbott DW , Finn RD , Spribille T . 2021. Predicted input of uncultured fungal symbionts to a lichen symbiosis from metagenome‐assembled genomes. Genome Biology and Evolution 13: 18. 10.1093/gbe/evab047 PMC8355462 33693712

[nph18780-bib-0142] Tarakhovskaya ER , Maslov YI , Shishova MF . 2007. Phytohormones in algae. Russian Journal of Plant Physiology 54: 163–170.

[nph18780-bib-0143] du Toit SF , Bentley J , Farrant JM . 2021. NADES formation in vegetative desiccation tolerance: prospects and challenges. In: Verpoorte R , Witkamp GJ , Choi YH , eds. Eutectic solvents and stress in plants. London, UK: Academic Press–Elsevier Science, 225–252.

[nph18780-bib-0144] Trembley ML , Ringli C , Honegger R . 2002. Morphological and molecular analysis of early stages in the resynthesis of the lichen *Baeomyces rufus* . Mycological Research 106: 768–776.

[nph18780-bib-0145] Tschermak‐Woess E . 1978. *Myrmecia reticulata* as a phycobiont and free‐living – free‐living *Trebouxia* – the problem of *Stenocybe septata* . The Lichenologist 10: 69–79.

[nph18780-bib-0146] Tunnacliffe A , Wise MJ . 2007. The continuing conundrum of the LEA proteins. Naturwissenschaften 94: 791–812. 17479232 10.1007/s00114-007-0254-y

[nph18780-bib-0147] Unal D , Senkardesler A , Sukatar A . 2008. Abscisic acid and polyamine contents in the lichens *Pseudevernia furfuracea* and *Ramalina farinacea* . Russian Journal of Plant Physiology 55: 115–118.

[nph18780-bib-0148] Vivas M , Sacristán M , Legaz ME , Vicente C . 2010. The cell recognition model in chlorolichens involving a fungal lectin binding to an algal ligand can be extended to cyanolichens. Plant Biology 12: 615–621. 20636904 10.1111/j.1438-8677.2009.00250.x

[nph18780-bib-0149] Wang XY , Wei XL , Luo H , Kim JA , Jeon HS , Koh YJ , Hur JS . 2010. Plant hormones promote growth in lichen‐forming fungi. Mycobiology 38: 176–179. 23956650 10.4489/MYCO.2010.38.3.176 PMC3741542

[nph18780-bib-0150] Wang Y , Han KS , Wang XY , Koh YJ , Hur JS . 2009. Effect of ribitol and plant hormones on aposymbiotical growth of the lichen‐forming fungi of *Ramalina farinacea* and *Ramalina fastigiata* . Mycobiology 37: 28–30. 23983503 10.4489/MYCO.2009.37.1.028 PMC3749451

[nph18780-bib-0151] Wang YY , Liu B , Zhang XY , Zhou QM , Zhang T , Li H , Yu YF , Zhang XL , Hao XY , Wang M *et al*. 2014. Genome characteristics reveal the impact of lichenization on lichen‐forming fungus *Endocarpon pusillum* Hedwig (Verrucariales, Ascomycota). BMC Genomics 15: 34. 24438332 10.1186/1471-2164-15-34 PMC3897900

[nph18780-bib-0152] Whiton JC , Lawrey JD . 1984. Inhibition of crustose lichen spore germination by lichen acids. Bryologist 87: 42–43.

[nph18780-bib-0153] Yoshimura I , Kurokawa T , Yamamoto Y , Kinoshita Y . 1993. Development of lichen thalli *in vitro* . Bryologist 96: 412–421.

[nph18780-bib-0154] Yoshino K , Yamamoto K , Hara K , Sonoda M , Yamamoto Y , Sakamoto K . 2019. The conservation of polyol transporter proteins and their involvement in lichenized Ascomycota. Fungal Biology 123: 318–329. 30928040 10.1016/j.funbio.2019.01.006

[nph18780-bib-0155] Yoshino K , Yamamoto K , Masumoto H , Degawa Y , Yoshikawa H , Harada H , Sakamoto K . 2020. Polyol‐assimilation capacities of lichen‐inhabiting fungi. The Lichenologist 52: 49–59.

